# Core Measure Set for Patient Safety in Perioperative Care: A Clinical Practice-Oriented Consensus Study

**DOI:** 10.3389/ijph.2026.1609159

**Published:** 2026-03-02

**Authors:** Ana Beatriz Nunes, José Pedro Teixeira, Andreia Leite, Willemijn Schäfer, Claudia Valli, Ismael Martínez-Nicolas, Margarida Paixão, Ayshe Seyfulayeva, Daniel Treviño Abelenda, Anna Rodríguez, Pedro Casaca Carvalho, Anita Heideveld-Chevalking, Yvette Emond, Kaja Põlluste, Hiske Calsbeek, Daniel Arnal-Velasco, Adam Žaludek, Pascal Garel, Cordula Wagner, Oliver Groene, Carola Orrego, Paulo Sousa, SAFEST Scientific Advisory Group, SAFEST Scientific Executive Group, SAFEST Consortium

**Affiliations:** 1 NOVA National School of Public Health, Public Health Research Centre, Comprehensive Health Research Center (CHRC), NOVA University Lisbon, Lisbon, Portugal; 2 Department of Epidemiology, National Institute of Health Doctor Ricardo Jorge, Lisbon, Portugal; 3 Northwestern Quality Improvement, Research & Education in Surgery, Department of Surgery, Northwestern University, Chicago, IL, United States; 4 Avedis Donabedian Research Institute, Barcelona, Spain; 5 Universitat Autonoma de Barcelona, Barcelona, Spain; 6 Spanish Anaesthesia and Reanimation Incident Reporting System (SENSAR), Alcorcón, Spain; 7 Foundation for Healthcare Training & Research of the Region of Murcia, Institute for Biomedical Research of Murcia, FFIS-IMIB Pascual Parrilla, Murcia, Spain; 8 Public Health Unit Francisco George, Unidade Local de Saude Santa Maria, Lisbon, Portugal; 9 Radboud University Medical Center (Operating Theatres, Centre of Acute and Intensive Care), Nijmegen, Netherlands; 10 IQ Health Scientific Department, Radboud University Medical Center, Nijmegen, Netherlands; 11 Institute of Clinical Medicine, University of Tartu, Tartu, Estonia; 12 Department of Anaesthesia and Reanimation, Hospital Universitario Fundacion Alcorcon, Alcorcón, Spain; 13 Third Faculty of Medicine, Department of Public Health, Charles University, Prague, Czechia; 14 Spojená Akreditační Komise (SAK), Prague, Czechia; 15 European Hospital and Healthcare Federation (HOPE), Brussels, Belgium; 16 Netherlands Institute for Health Services Research (Nivel), Utrecht, Netherlands; 17 Amsterdam University Medical Center, Amsterdam, Netherlands; 18 Optimedis AG, Hamburg, Germany; 19 Universitat Witten/Herdecke, Witten, Germany; 20 Red de Investigacion en Cronicidad Atencion Primaria y Prevencion y Promocion de la Salud, Barcelona, Spain

**Keywords:** patient safety, perioperative care, core measure set, core outcome set, quality indicators, performance measures, outcome assessment, routine care evaluation

## Abstract

**Objectives:**

To develop a consensus-based EU-wide Core Measure Set (CMS), including patient-reported measures, for evaluating perioperative patient safety in routine adult care.

**Methods:**

A four-phase approach was applied: 1) identification of candidate structure, process, and outcome measures through three literature reviews; 2) synthesis into an initial list via deduplication and merging; 3) consensus process using a two-round modified eDelphi technique, with subject-matter and lived-experienced experts rating the importance and feasibility of each measure (consensus: ≥75% rating 7–9, ≤15% rating 1–3), followed by an online Consensus Conference addressing measures highly valued by patients but lacking feasibility consensus; 4) refinement of the CMS for patient safety in perioperative care (CMS-PSPC).

**Results:**

Out of 9,717 records identified, 340 studies were included, yielding 1,305 measures. After refinement, 247 measures were consolidated; 84 reached consensus, and ten more were added via the Consensus Conference. The final CMS-PSPC comprised 76 measures: 22 outcome, 18 process, and 36 structure measures.

**Conclusion:**

The CMS-PSPC provides a standardised, patient-centred framework for evaluating, monitoring and benchmarking perioperative patient safety in routine clinical care, supporting EU-wide quality improvement efforts.

## Introduction

Unsafe healthcare is a major public health problem, resulting in approximately 64 million disability-adjusted life-years, over 3 million deaths annually, and a 15%–20% decrease in patient quality of life [1]. In the European Union (EU), 8%–12% of hospitalised patients experience adverse events, many preventable [2–5]. Surgical-related unsafe care, one of the most prevalent in-hospital adverse events, primarily occurs outside the operating room, causing around half a million preventable deaths each year [6–8]. The economic burden of unsafe care is considerable, amounting to 17–38 billion Euros in direct costs in Europe and 1-2 trillion USD in indirect costs annually, negatively impacting economic growth and productivity [1, 9].

Measurement is crucial for patient safety, enabling the identification of adverse event trends, priority setting and resource allocation, evaluation of safety interventions, and assessment of healthcare service performance [10–12]. However, accurate and reliable measurement of adverse events remains challenging due to the limited monitoring capacity of healthcare systems [12]. Recognising this challenge, in 2015, the National Patient Safety Foundation recommended the establishment of a “common set of safety metrics” to advance patient safety. Similarly, the World Health Organization’s Global Patient Safety Action Plan (2021–2030) urged governments to develop “indicators for patient safety aligned with global patient safety targets” [13, 14]. Despite these initiatives, previous efforts in perioperative care have primarily focused on research settings or outcome measures alone [15–19], highlighting a gap in standardised, comprehensive, patient-centred measurement of perioperative safety in clinical practice.

Core Outcome Sets (COS) have emerged as a methodologically robust approach to standardise outcome measurement and reporting for specific clinical conditions [20, 21]. While initially developed for research, COS are increasingly used in clinical guidelines, audits, and quality improvement initiatives, reflecting their value in promoting patient-centred and value-based healthcare [20, 22, 23]. Nevertheless, focusing solely on outcomes is insufficient for a comprehensive assessment of patient safety throughout the perioperative journey.

To address this limitation, a more comprehensive framework that integrates outcome, process, and structure measures is needed. The Core Quality Measures Collaborative supports this approach, emphasising the importance of diverse measure types while maintaining outcomes as key indicators of overall healthcare quality [24]. This broader approach aligns with Donabedian’s quality of care conceptual model, which states that appropriate structures and processes increase the likelihood of achieving favourable care outcomes [10, 25].

A standardised Core Measure Set (CMS) encompassing structure, process, and outcome measures is thus essential. Such a CMS provides a scientifically robust, patient-centred framework for quality assessment [24], enabling comprehensive evaluation and monitoring of patient safety across clinical care, research, and healthcare management, as well as benchmarking across services, countries, and time.

This study aimed to develop a consensus-based EU-wide Core Measure Set, including patient-reported measures, for evaluating patient safety in routine perioperative healthcare services for adult patients.

## Methods

### Study Design

**FIGURE 1:**
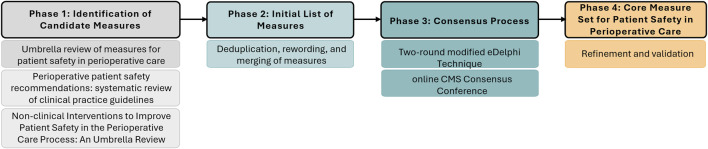
Core Measure Set for Perioperative Patient Safety: study phases (European Union, 2022–2023).

The development of the CMS for Patient Safety in Perioperative Care (CMS-PSPC) followed a multimethod approach comprising four sequential phases: 1) identification of candidate structure, process, and outcome measures through a series of literature reviews; 2) synthesis of available measures into an initial list of measures; 3) a consensus process combining a two-round modified eDelphi technique with an online consensus conference; and 4) refinement and final agreement on the CMS-PSPC (Figure 1).

This study was conducted as part of the ‘Improving Quality and Patient SAFEty in Surgical Care through STandardisation and Harmonisation of Perioperative Care in Europe’ (SAFEST) project [26]. While it builds upon foundational SAFEST studies, this manuscript reports the unique results of the CMS-PSPC development process, which have not been previously published. Although the study adhered to the established COS methodology for development and reporting [20, 21, 27], its scope was expanded to rigorously develop a comprehensive CMS. Accordingly, this study was prospectively registered in the COMET Database [28] as a COS; the terminology was subsequently updated to CMS to reflect the inclusion of structure and process measures alongside outcomes, while maintaining the registered methodology.

### Phase 1: Identification of Candidate Measures

The primary source for the initial list of measures was an umbrella review of measures for patient safety in perioperative care in surgical adult patients [29]. This was complemented by measures identified in a systematic review of clinical practice guidelines and an umbrella review of nonclinical interventions to improve perioperative patient safety, both conducted within the SAFEST project [30, 31]. Within this context, nonclinical interventions are defined as organisational, managerial, or behavioural strategies implemented independently of direct clinical encounters between healthcare professionals and patients.

#### Umbrella Review of Measures for Patient Safety in Perioperative Care

The protocol for the umbrella review of measures for patient safety in perioperative care has been prospectively registered in PROSPERO (CRD42022362921) [29].

To ensure comprehensive coverage of the relevant literature, the following electronic databases were searched: PubMed, Scopus, Web of Science (Core Collection), Embase, The Cochrane Library (Cochrane Database of Systematic Reviews), Cumulative Index to Nursing and Allied Health Literature, and COMET Initiative database. This was supplemented by reference mining of eligible systematic reviews. Detailed search strategies are provided in Supplementary Material 1.

Inclusion criteria for the umbrella review encompassed quantitative, qualitative, and mixed-methods systematic reviews focusing on measures developed within the context of CMS; additionally, studies were considered eligible if they employed methodologies aimed at identifying pertinent measures for routine care, audits, or clinical trials. No limitations were imposed on language or timeframe. Exclusion criteria included systematic reviews lacking accessible lists of measures and those not developed within the context of CMS or similar methodologies.

After calibration, two researchers (ABN, JPT/MP) independently conducted title and abstract screening to identify eligible systematic reviews, followed by full-text screening (ABN, JPT). Screening was facilitated using Rayyan [32]. Data extraction was performed by one researcher (ABN) using a pilot-tested standardised data extraction form, which was then reviewed by another researcher (JPT). For each study, citation details, review type, and methodology used to generate the initial list of measures were extracted. Within a given study, measures’ names, definitions, and measurement instruments were extracted verbatim. Discrepancies regarding screening and data extraction between authors were resolved by consensus.

The extracted measures were synthesised and categorised by perioperative period (preoperative, intraoperative, postoperative and mixed), Donabedian’s quality of care conceptual model (structure, process, outcome) [10], and measure domain. Categorisation followed an inductive-deductive approach. Measures referring to the same or very similar underlying constructs were categorised under a common term. Categorisation of each verbatim measure definition to a measure name, and measure name to a measure domain was performed by one researcher (ABN/JPT) and reviewed by another researcher (WS/AL/CO). Discrepancies were resolved by consensus or by a third appraiser if needed (PS).

#### Complementary Systematic and Umbrella Reviews

The detailed methodologies of the reviews “Perioperative patient safety recommendations: systematic review of clinical practice guidelines” and “Non-clinical Interventions to Improve Patient Safety in the Perioperative Care Process: An Umbrella Review” are reported elsewhere [30, 31]. Both reviews provided additional measures beyond those identified in the primary umbrella review. Data extraction (CV, DTA, AR) followed a consistent approach across all reviews.

### Phase 2: Initial List of Measures

The candidate list of perioperative patient safety measures identified in Phase 1 literature reviews was refined iteratively by removing duplicates, rewording, and merging similar measures to ensure suitability for implementation in the modified eDelphi technique. The following criteria were considered: similarity of underlying constructs, avoidance of repetition, clarity of measures, applicability to a wide range of surgical procedures, adherence to patient safety principles and concepts, support advancing patient safety improvement goals, and promotion of patient engagement in patient safety. Each measure was initially assessed by one researcher (ABN) and subsequently reviewed by two additional researchers (WS/CV/AL, CO/PS). Discrepancies were resolved through consensus-based discussions. This process yielded the initial list of measures assessed in the subsequent modified eDelphi technique (Phase 3).

### Phase 3: Consensus Process

#### Consensus Panel Selection and Recruitment

The Consensus Panel comprised experts from a previous SAFEST project study and specialists in CMS development and outcomes research [33, 34]. A stratified purposive sampling approach [35] was employed to ensure a balanced and heterogeneous expert panel across multiple dimensions: subject-matter knowledge in patient safety, perioperative care or outcomes research, stakeholder groups, professional backgrounds, healthcare settings, and sociodemographic characteristics (e.g., gender, geography). Given the importance of patients involvement in CMS development, patient representatives were included to incorporate lived experience-based expertise [20, 34]. The eligibility criteria required experts to be adults who could complete online surveys in English. Participation in the two-round modified eDelphi technique was not compulsory for participation in the CMS Consensus Conference.

Further details on the selection and recruitment methodologies for the Consensus Panel, as well as the methods employed in the modified eDelphi technique, are available elsewhere [33, 34].

#### Modified eDelphi Technique

The two-round modified eDelphi technique was conducted from 24 February to 2 May 2023 using the Welphi platform [36, 37]. Round 1 spanned 3 weeks, round 2 two weeks. Three reminders were sent to non-respondents in each round.

In both rounds, experts rated each measure from the initial list of measures for importance and feasibility (defined as the ease of measuring a quality indicator accurately) using a fully labelled 9-point Likert scale (1: “Not important at all”/“Extremely difficult to measure;” 9: “Critical for inclusion”/“Extremely easy to measure”) [38]. Importance and feasibility were assessed to ensure selected measures were relevant, practical and implementable in real-world clinical settings [38, 39]. An “I don’t know” option was available, acknowledging the panel’s diverse composition. Experts could provide open-text comments to justify their ratings.

Consensus was defined *a priori* for both importance and feasibility using percentage agreement. A measure achieved consensus for inclusion in the preliminary final list of measures if ≥75% of experts rated it as 7–9, and no more than 15% rated it as 1–3. Conversely, consensus for exclusion was reached if ≥75% of experts rated a measure 1–3 and no more than 15% rated it 7–9. These criteria were consistently applied across both eDelphi rounds [34].

After each round, experts received a summary report with descriptive statistics for each measure (e.g., combined 7%–9% agreement, average rating and standard deviation), and anonymised expert comments. Qualitative feedback from round 1 informed the clarification, merging, and modification of the measures’ names and definitions for round 2.

Given the substantial number of measures within the initial list of measures, those that reached consensus for inclusion without significant comments suggesting revisions were directly added to the preliminary final list without undergoing re-rating in round 2. Measures that achieved consensus on only one dimension (importance or feasibility) in round 1 without additional comments were re-rated solely on the dimension lacking consensus. All other measures were retained for round 2.

Statistical analyses were conducted using Microsoft Excel.

#### CMS Consensus Conference

The final phase of the consensus process for the CMS-PSPC was a 2.5-h online conference held on 11 May 2023 using the group Delphi methodology. This structured method uses real-time interaction to facilitate consensus and highlight areas of dissent among experts [40].

After the presentation of round 2 results from the modified eDelphi technique, experts, guided by SAFEST researchers (ABN, JPT, AS, PCC, CV, AR, CO, WS, IMN, PS), discussed 25 measures that had achieved consensus on importance but lacked consensus on feasibility. These measures fell into two categories: 1) those rated highly important by ≥80% of patients and deemed feasible by ≥60% of the overall expert panel; and 2) those unanimously rated as highly important by patients, regardless of feasibility scores.

In randomly assigned breakout sessions, subgroups identified barriers hindering the feasibility of preassigned measures and subsequently proposed facilitators and solutions. Each subgroup then collaboratively scored these measures on a 9-point Likert scale (1: Not important at all; 9: Critical for inclusion) regarding their importance for inclusion in the preliminary final list, irrespective of feasibility constraints. Divergent opinions were acknowledged and justified. After small group discussions, the plenary session reconvened for subgroup presentations and open feedback from other participants. Measures scoring 8 or 9 and those noted as highly important through participant comments were included in the preliminary final list of measures.

### Phase 4: Core Measure Set for Patient Safety in Perioperative Care

Following the CMS Consensus Conference, the preliminary final list of measures was emailed to panel experts for further feedback.

During and following the CMS Consensus Conference, experts raised concerns about the feasibility of implementing the number of measures included in the preliminary final list within real-world clinical practice settings. Therefore, the research team (ABN, CV, AR, CO, WS, IMN, PS) undertook further analysis to synthesise measures with similar underlying constructs and to improve the wording of measure names and definitions. To process and structure measures, concise name measures were assigned while preserving the definitions established in preceding documentation. To enhance the practical application and usability of the CMS, the final list of measures was organised in a tiered manner [1]: *Critical and feasible measures*: those rated between 8-9 for importance and between 7–9 for feasibility by at least 75% of the experts; and [2] *Important and feasible measures*: those rated between 7–9 for both importance and feasibility by at least 75% of the experts. For outcome measures, a third category was defined as important measures, referring to those that, despite lacking a feasibility consensus, were highly valued by patients and achieved consensus on their importance among all experts.

The resulting set of measures, comprising the CMS-PSPC, was then shared with the expert panel and the SAFEST’s Scientific Executive Group (SEG) [26] for final review and validation.

### Ethical Considerations

The SAFEST studies received ethical approval by IDIAP Jordi Gol’s Research and Ethics Committee (CEIm code 22/146-P) [26]. All eDelphi panel participants provided written informed consent prior to participation. For the Consensus Conference, oral informed consent was obtained for audio recording.

## Results

### Identification of Candidate Measures

#### Umbrella Review of Measures for Patient Safety in Perioperative Care

After deduplication, 1925 records were screened, with only 11 studies meeting the predefined inclusion criteria following full-text screening (Supplementary Material 2). All included reviews were published between 2018 and 2022, and most (n = 9) in anaesthesiology journals.

From the 11 included studies [15–18, 41–47], 588 measures were extracted, including 114 Patient-Reported Outcome Measures (PROMs)/Patient-Reported Experience Measures (PREMs). Most measures were: outcome measures (n = 433); primarily related to the postoperative period (n = 434); and categorised within the domains of adverse events (n = 187), compliance with safety practices (n = 124), and functional status (n = 85).

#### Complementary Systematic and Umbrella Reviews

In the systematic review “Perioperative patient safety recommendations: systematic review of clinical practice guidelines,” 267 studies yielded 77 measures. Five measures were identified as novel, not previously identified in the primary umbrella review, and added to the candidate list of measures.

In the umbrella review “Non-clinical interventions to improve patient safety in the perioperative care process,” 62 studies were included, yielding 640 measures. Of these, 32 measures were unique measures, and were thus added to the candidate list of measures.

**FIGURE 2:**
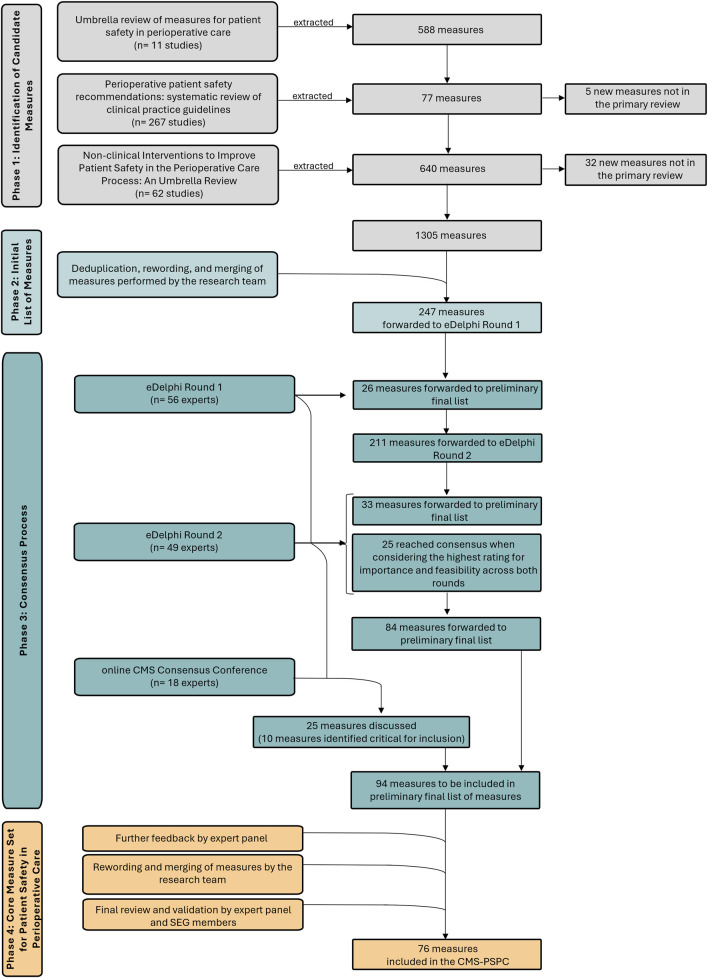
Core Measure Set for Patient Safety in Perioperative Care: development process and findings overview (European Union, 2022–2023). Summary of the four-phase multimethod approach used to develop the Core Measure Set for Patient Safety in Perioperative Care, illustrating the attrition of measures from 1,305 candidates identified through literature reviews to a final set of 76 measures, selected through expert consensus and iterative refinement.

The three reviews collectively screened 9,717 records, of which 340 studies met the inclusion criteria yielding 1,305 measures extracted (Figure 2).

### Initial List of Measures

The 1,305 extracted measures were refined through deduplication, rewording, and merging by the research team, resulting in a consolidated list of 247 measures (Figure 2; Supplementary Material 3). The initial list of measures predominantly comprised outcome measures, measures related to the postoperative period and categorised within the domains of adverse events, compliance with safety practices, and functional status.

### Consensus Process

#### Modified eDelphi Technique

Fifty-six experts participated in round 1 and 49 in round 2 of the modified eDelphi technique, resulting in a retention proportion of 87.5%. The consensus panel included diverse targeted stakeholder groups, with over 76.5% being healthcare professionals and approximately 10.0% being patients. The sociodemographic characteristics of the participants in both eDelphi rounds are detailed in Table 1.

**TABLE 1:** Sociodemographic characteristics of participants in rounds 1 and 2 of the eDelphi survey. (European Union, 2022–2023).

	eDelphi survey round			
	Round 1		Round 2	
Sociodemographic characteristic	n	%	n	%
**Gender**	n = 56		n = 49	
Female	22	39.3%	20	40.8%
Male	31	55.4%	26	53.1%
Non-binary	0	0.0%	0	0.0%
Prefer not to answer	3	5.4%	3	6.1%
**Age group**	n = 56		n = 49	
18–34	2	3.6%	2	4.1%
35–54	22	39.3%	19	38.8%
55–74	29	51.8%	25	51.0%
75+	1	1.8%	1	2.0%
Prefer not to answer	2	3.6%	2	4.1%
**Highest level of education completed**	n = 56		n = 49	
Postgraduate education	50	89.3%	44	89.8%
Tertiary (higher) education	4	7.1%	3	6.1%
Secondary school	2	3.6%	2	4.1%
Primary school	0	0.0%	0	0.0%
None/Incomplete primary school/Prefer not to answer	0	0.0%	0	0.0%
**Role**	n = 56		n = 49	
Healthcare professional	43	76.8%	40	81.6%
Profession				
Medical doctor	33	76.7%	30	75.0%
Nurse	5	11.6%	5	12.5%
Quality expert	2	4.7%	2	5.0%
Hospital manager	1	2.3%	1	2.5%
Physical therapist	1	2.3%	1	2.5%
Other	1	2.3%	1	2.5%
Prefer not to answer	0	0.0%	0	0.0%
Area of expertise				
Anaesthesiology	20	46.5%	18	45.0%
Surgery	9	20.9%	9	22.5%
Primary care	3	7.0%	3	7.5%
Public health	3	7.0%	3	7.5%
Haematology	2	4.7%	2	5.0%
Rehabilitation	1	2.3%	1	2.5%
Other	5	11.6%	4	10.0%
Geriatrics	0	0.0%	0	0.0%
Prefer not to answer	0	0.0%	0	0.0%
Patients and/or patient representative	6	10.7%	5	10.2%
Governmental agency representative	3	5.4%	1	2.0%
Methodologist/Researcher	2	3.6%	1	2.0%
Policymaker	1	1.8%	1	2.0%
Private sector representative	1	1.8%	1	2.0%
Prefer not to answer	0	0.0%	0	0.0%

In round 1, 247 measures were assessed, with 40 (16.2%) achieving consensus for inclusion based on importance and feasibility. Of these, 26 measures were directly added to the preliminary final list, while 12 were reworded and re-rated in round 2. Two measures were added, 11 merged, and four removed based on expert input. In round 2, 211 unique measures were assessed, with 126 rated for importance and 207 for feasibility. Among these, 33 (15.6%) achieved consensus for inclusion in the preliminary final list. An additional 25 measures met the consensus for inclusion when considering their highest importance and feasibility ratings across both rounds (Figure 2, Supplementary Material 3).

Overall, 84 measures (34.0%) obtained consensus for inclusion based on both importance and feasibility (Figure 2, Supplementary Material 3). No measures reached consensus for exclusion in either round. In both rounds, importance consistently showed a higher consensus for inclusion than feasibility across subgroups of Donabedian’s quality of care conceptual model and perioperative periods. The intraoperative period achieved a higher consensus for inclusion than the other perioperative periods for both importance and feasibility. Structure measures generally obtained the highest consensus for inclusion.

A comprehensive overview of the results obtained in this modified eDelphi technique can be found in Dinis-Texeira et al. [34].

#### CMS Consensus Conference

The CMS Consensus Conference was attended by 18 panel experts, all of whom were healthcare professionals, 13 SAFEST partners, and four SEG members. Of the 25 measures discussed, 19 were outcome measures, five were process measures, and one was a structure measure (Supplementary Material 3); most measures (n = 11) regarded the postoperative period.

Experts identified several barriers and facilitators. For process measures, key barriers included the limited availability of patient education materials tailored to varying health literacy levels and the challenge of measuring patient information-related metrics, as information delivered does not ensure understanding. Experts also noted the risk of reporting bias and the difficulty in accurately capturing certain process measures. Efficient electronic administrative systems, Electronic Health Records (EHR), and streamlined workflows were recognised as important facilitators. For outcome measures, barriers included insufficient human resources for systematic assessment and the burden placed on patients to complete PROM surveys. As facilitators, experts emphasised efficient interoperable communication systems for inter-provider data sharing, EHR with mandatory structured fields for streamlined data recording, online surveys, and smartphone applications to monitor patients’ physical activity.

During the subgroups and plenary discussions, experts identified 10 measures as critical for inclusion in the preliminary final list of measures (Figure 2, Supplementary Material 3).

### CMS-PSPC

**FIGURE 3:**
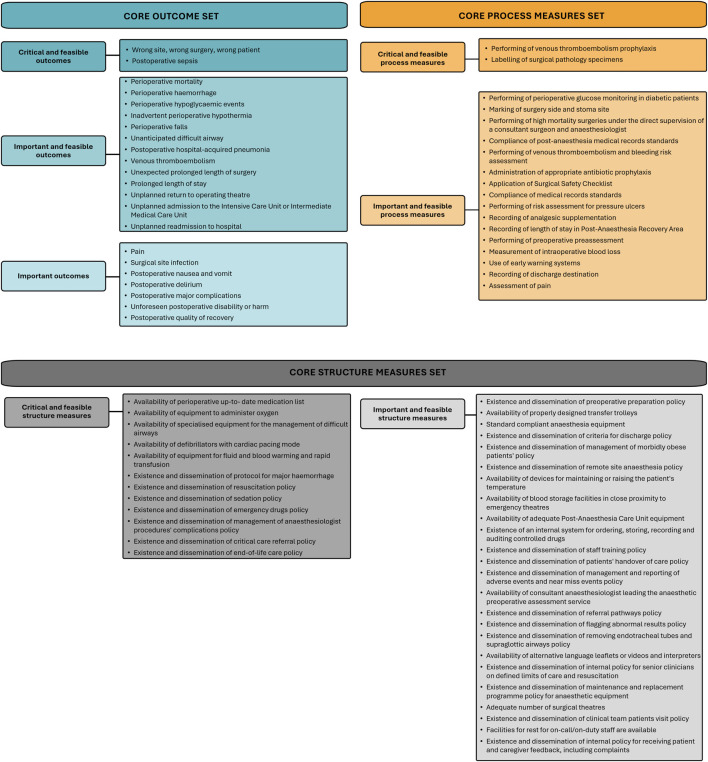
Core Measure Set for Patient Safety in Perioperative Care: Included Outcome, Process and Structure Measures (European Union, 2022–2023). The Core Measure Set for Patient Safety in Perioperative Care, comprising 76 measures, is presented according to Donabedian’s quality of care conceptual model (outcome, process, and structure). The measures were tiered based on consensus ratings to reflect their relative importance and feasibility. This categorisation and tiered structure are intended to support flexible, context-specific selection of CMS subsets or prioritisation of measures for routine monitoring, benchmarking, and evaluation across diverse clinical settings.

Following the CMS Consensus Conference, four expert panel members provided additional feedback via email. From the preliminary final list of measures comprising 94 measures (Figure 2), the definitive CMS-PSPC was refined to 76 measures, spanning the entire perioperative period: 22 outcome measures, 18 process measures, and 36 structure measures (Figure 3 and Supplementary Material 4).

## Discussion

The CMS-PSPC comprises 76 measures that constitute a comprehensive, patient-centred framework for evaluating perioperative patient safety using structure, process, and outcome measures.

The development of this CMS followed a rigorous, multimethod iterative process aligned with the COMET guidance and compliant with the Core Outcome Set–STAndards for Development (COS-STAD) recommendations, the Core Outcome Set–STAndards for Reporting (COS-STAR) Statement, and methodological criteria for reporting Delphi studies [20, 21, 27, 36]. A modified eDelphi technique was applied to capture the perspectives and expertise from an EU-wide, multi-stakeholder and cross-disciplinary panel, including patients. The process employed a 9-point Likert scale and stringent consensus criteria to support a more selective inclusion of measures, an approach also adopted in other studies [48–53]. Feasibility was explicitly considered during the consensus process to improve its applicability in real-world healthcare settings, and the CMS broadened its scope beyond outcomes to include process and structure measures, enabling a comprehensive assessment of perioperative patient safety across all essential dimensions [10]. Similar methodological approaches have been employed in previous CMS and COS development efforts [54–56].

Despite successfully reducing the initial 247 measures to 76, the CMS-PSPC remains substantial, challenging its implementation in healthcare services. To mitigate this, two strategies are proposed: a) selecting a context-relevant subset of the CMS (Core Outcome Set, Core Process Measure Set, and Core Structure Measure Set) tailored to specific needs of the implementing healthcare service; and b) prioritising measures in resource-limited settings using the classification of critical or important measures. Healthcare services can, thus, adopt a modular implementation strategy tailored to their specific clinical context and available resources. For example, facilities with limited data-collection infrastructure may initially focus on foundational “critical and feasible structure measures” (e.g., existence and dissemination of protocol for major haemorrhage) assessed through routine administrative audits. Conversely, well-resourced healthcare services with established structure and process workflows can leverage advanced EHR systems to automate the monitoring of “important and feasible outcome measures” (e.g., perioperative haemorrhage), integrating these into clinical dashboards; this enables surgical teams to benchmark against EU-wide safety standards without additional manual data entry. Such a tiered approach facilitates targeted patient safety interventions, minimises administrative burden, and remains adaptable to diverse clinical settings. Although developed for the EU context, this flexible, modular design can also be implemented in non-EU healthcare systems, where it can be adapted to local resources and priorities.

The CMS-PSPC includes 22 outcome measures, a number consistent with other COS developed for routine care, which typically encompass more outcomes than research-focused sets [23]. These include mortality, adverse event-related, and resource use-related outcomes, commonly included in COS for surgical care or routine clinical settings, highlighting their importance across clinical conditions and healthcare contexts [19, 23, 52, 56]. While postoperative quality of recovery and unforeseen postoperative disability or harm were prioritised in the CMS-PSPC, other life-impact-related-outcomes were not included. The absence of outcomes related to social functioning, emotional wellbeing, and quality of life may reflect the underrepresentation of patient views, as the expert panel primarily consisted of healthcare professionals and no patients attended the CMS Consensus Conference. Research has shown that the inclusion of life-impact-related outcomes correlates with the degree of patient participation [23]. This gap may also reflect different priorities, as patients value long-term wellbeing, including emotional, social, and functional recovery, while healthcare professionals focus on system-level factors, such as protocol adherence and clinical outcomes within their control [57]. As a result, the CMS-PSPC likely underrepresents life-impact-related-outcomes, potentially limiting its ability to fully capture patient-centred aspects of perioperative safety and long-term wellbeing. To mitigate this gap, users may supplement the CMS with patient-prioritised measures when evaluating perioperative safety.

Structure measures showed highest consensus for inclusion, contrasting with prior research on patient safety that reported a lack of prioritisation for such measures [55]. This divergence may reflect the expert panel’s recognition of the foundational role that structural elements play in facilitating safe perioperative care as well as their comparatively easier assessment [10, 25].

### Limitations

The feasibility of implementing outcome measures emerged as a challenge during the consensus process, with some experts emphasising the need for specific measurement instruments and others noting the burden associated with data collection. Low feasibility ratings (e.g., postoperative nausea and vomit, preoperative frailty assessment) may reflect varied interpretations of the term or the limited representation of outcomes research experts on the panel. Overcoming these issues will require identifying appropriate measurement instruments and leveraging high-quality health information systems [39].

The exclusive use of the English language throughout the consensus process may have hindered the participation of non-native English-speaking experts, as noted in other studies [50, 58].

Despite efforts to ensure balanced representation across gender, geography, and stakeholder groups, challenges in achieving equitable distribution likely influenced the consensus on the CMS-PSPC. Although patients constituted 10% of participants in the modified eDelphi technique, consistent with other studies, and despite the CMS Consensus Conference focusing on measures highly valued by patients, their absence from the Conference underscores the need for enhanced engagement strategies [20] and for consensus processes that explicitly integrate patient-prioritised outcomes.

### Implications for Practice and Policy

By providing a standardised framework, the CMS-PSPC offers a robust tool for benchmarking patient safety performance across hospitals and health systems over time, facilitating the development of best practices, and driving quality improvement initiatives across the EU.

From a clinical perspective, the CMS-PSPC empowers healthcare professionals to compare practices and identify opportunities for continuous improvement in patient safety. For healthcare managers, systematically evaluating perioperative patient safety practices provides actionable insights, driving strategic improvements in patient outcomes and experiences by focusing on areas of subpar performance rather than assigning blame. At the policy level, the CMS-PSPC can inform payment and regulatory reforms, supporting evidence-driven policies that incentivise high-quality care and align healthcare services with the highest standards of patient safety.

### Future Work

Future research should focus on developing effective strategies to integrate the CMS-PSPC assessments into routine clinical practice. A key next step is creating a practical handbook to guide clinical teams, IT departments, and healthcare managers in selecting appropriate measurement instruments and implementing IT-Ready Sets [59, 60]. Such a handbook would streamline data collection through standardised coding and offer advice on targeted implementation strategies that recognise organisational culture as a critical enabler of sustained use. Finally, evaluating the impact of CMS implementation on patient safety outcomes in diverse perioperative healthcare settings is vital for ensuring long-term effectiveness.

### Conclusion

Developed within the SAFEST project, the CMS-PSPC establishes a standardised, patient-centred framework for assessing perioperative safety, integrating structure, process, and outcome measures. While full implementation may pose challenges, its categorisation into subsets and assessing importance allows for adaptable, context-specific implementation across healthcare services. By incorporating patient perspectives, supporting the evaluation of quality improvement initiatives, and enabling benchmarking across EU healthcare services, the CMS-PSPC has the potential to enhance surgical outcomes, patient safety, and patient experiences, driving system-wide improvements in perioperative care.

## Group Members of SAFEST Scientific Advisory Group, SAFEST Scientific Executive Group, SAFEST Consortium

Aamer Ahmed, Fragkiskos Angelis, Catarina Baptista, Metaxia Bareka, Kateryna Bielka, Mercedes Bilbao, Federico Bilotta, Elvira Bisbe, Hans D de Boer, Dialina Brilhante, Pedro Carrascal, Pedro Delgado, Zsuzsanna Farkas-Pall, Aidan Fowler, Loredana Gigli, Helen Haskell, Arvid Steinar Haugen, Jan Hofland, Beverley Hunt, Ib Jammer, Janek Kapper, Marion van der Kolk, Natasa Kovac, Susana Lorenzo, Rui Malheiro, Xose Manuel Meijome, Jannicke Mellin-Olsen, Margaret Murphy, Maria Ntalouka, Marta Dora Ornelas, Maria Papadakaki, Danica Rotar Pavlic, Julien Picard, Marek Pietruszka, Benedikt Preckel, Finn Radktke, José Manuel Rodríguez, Narimantas Evaldas Samalavicius, Pedro Vieira dos Santos, Ed Schoemaker, Kawaldip Sehmi, Henriëtte Smid-Nanninga, Joel Starkopf, John Tansley, Zuzana Tomášková, Francesco Venneri, Matthias Weigl, Marja Boermeester, Katherine de Bienassis, Basil Matta, Barbara Kutryba, Basak Ceyda Meço, Thalia Manker, and Argyro Zoumprouli. Nuno Diogo, María del Mar Fernández, Pascal Garel, Oliver Groene, Anita Heideveld-Chevalking, Irene León, Peep Talving, Willemijn Schäfer, Marieke Voshaar, Maria Wittmann, and Adam Žaludek. Joaquim Bañeres, Ashish Bartakke, Genis Carrasco, Edoardo De Robertis, Neus Fabregas, Javier García-Silva, Mari Kangasniemi, Janne Kommusaar, Irene León, David Marx, Marie Nabbe, Janne Pühvel, Eva Romero-Garcia, Yolanda Sanduende-Otero, Victor Soria-Aledo, Joel Starkopf, Rosa Sunol, Marc Gibert, Sofía Carbonell, Sophie Wang, Helena Vall, Arta Leci, Nina van der Schoot, Lilian Van Tuyl, František Vlček, Marieke Voshaar, and Sandro Zamarian.
