# Supplementary material for: Core Measure Set for Patient Safety in Perioperative Care: A Clinical Practice-Oriented Consensus Study
Source: Int J Public Health. 2026 Mar 2;71:1609159. doi: 10.3389/ijph.2026.1609159 (PMC12989447; doi:10.3389/ijph.2026.1609159)

International Journal of Public Health

Title: **Core Measure Set for Patient Safety in Perioperative Care: A Clinical Practice-Oriented Consensus Study**

Supplementary file S2 - Flow diagram of the umbrella review "Umbrella review of measures for patient safety in perioperative care" (European Union, 2022-2023)

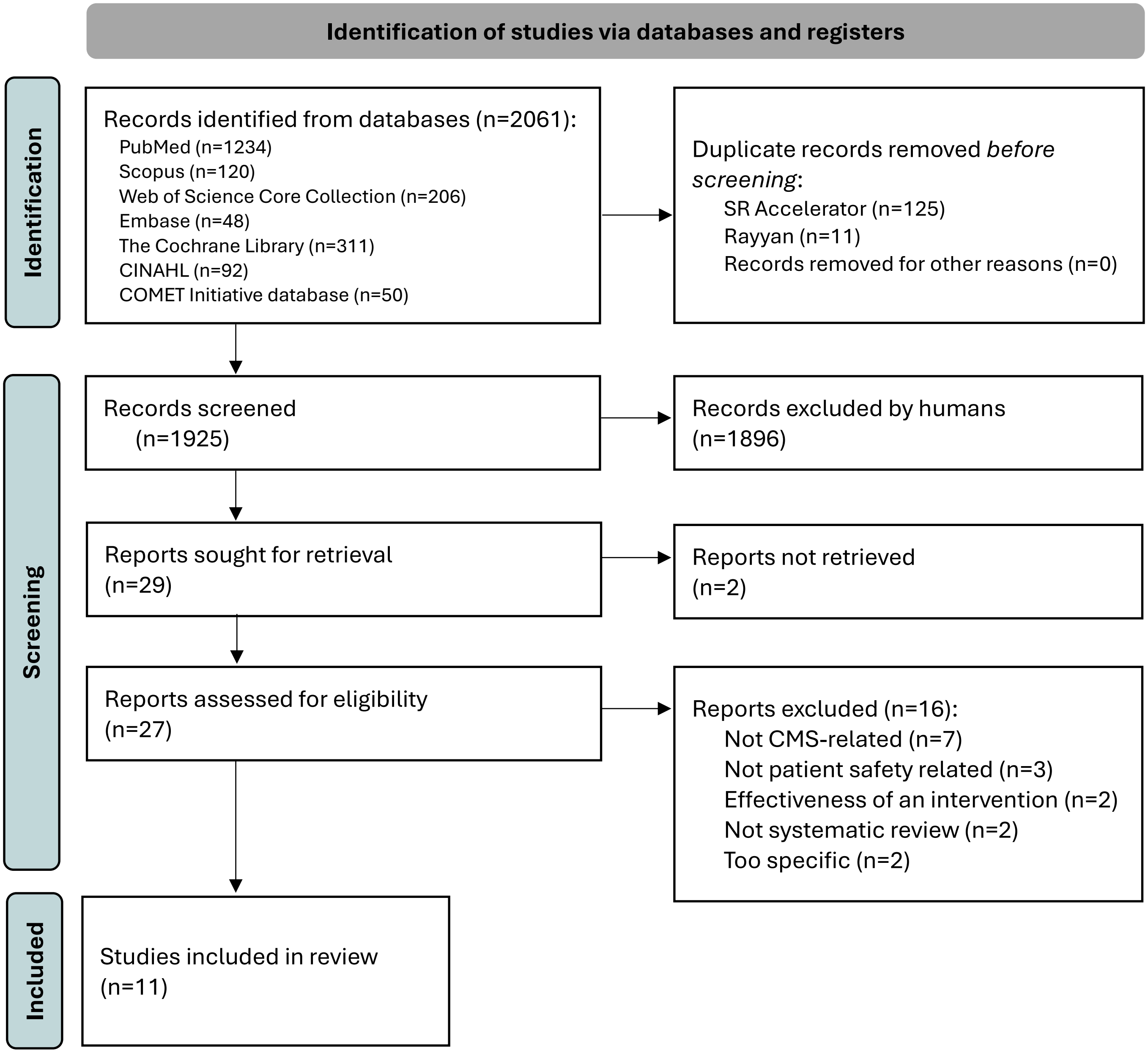

Supplement: Supplementary file 1 [file DataSheet2.pdf]
