# Supplementary material for: Core Measure Set for Patient Safety in Perioperative Care: A Clinical Practice-Oriented Consensus Study
Source: Int J Public Health. 2026 Mar 2;71:1609159. doi: 10.3389/ijph.2026.1609159 (PMC12989447; doi:10.3389/ijph.2026.1609159)
Supplement: Supplementary file 2 [file DataSheet4.pdf]

International Journal of Public Health

Title: **Core Measure Set for Patient Safety in Perioperative Care: A Clinical Practice-Oriented Consensus Study**

Supplementary file S4 - Core Measure Set for Patient Safety in Perioperative Care: description by outcome, process, and structure sets (European Union, 2023)

Core Outcome Set for Patient Safety in Perioperative Care

Category	Measure	Definition
**Critical and feasible outcomes**	Wrong site, wrong surgery, wrong patient	Surgery performed on the wrong side or site of the body, wrong surgical procedure performed, and surgery performed on the wrong patient.
	Postoperative sepsis	Life-threatening organ failure caused by the host's inappropriate response to infection, including septic shock, due to the surgical procedure and within 30 days after surgery.
**Important and feasible outcomes**	Perioperative mortality	Any death, regardless of cause, occurring within 30 and 90 days after surgery in or out of the hospital.
	Perioperative haemorrhage	Haemorrhage of an organ or structure that warrants an unanticipated blood transfusion due to the surgical procedure, during or after surgery. The term "unanticipated" implies that the transfusion of blood products was not included in the patient's initial treatment plan and was likely initiated as a consequence of unexpected situations such as sudden deterioration of the patient's health or unanticipated complications that occurred during the procedure.
	Perioperative hypoglycaemic events	Any in-hospital blood glucose levels under 50mg/dL occurring during the perioperative period.
	Inadvertent perioperative hypothermia	Any in-hospital drop of core body temperature to < 35.5°C, occurring during the perioperative period.
	Perioperative falls	Any in-hospital documented fall, occurring during the perioperative period.

	Unanticipated difficult airway	A conventionally trained anaesthesiologist experiences difficulty with facemask ventilation of the upper airway, difficulty with tracheal intubation, or both.<br>The term "unanticipated" implies that the difficult airway was not anticipated in the patient's initial assessment.
	Postoperative hospital-acquired pneumonia	Hospital-acquired pneumonia (pneumonia developing ≥ 48 hours after admission) or ventilator-associated pneumonia (pneumonia developing ≥ 48 hours after endotracheal intubation) occurring in patients who have undergone a surgical procedure.
	Venous thromboembolism	Pulmonary embolism or deep vein thrombosis following a surgical procedure.
	Unexpected prolonged length of surgery	Duration of the patient's surgery (minutes) superior to expected according to the complexity of the surgical procedure.
	Prolonged length of stay	Duration of patient's stay in the hospital from admission to discharge or death (in days) superior to expected (above median length of stay) due to the surgical procedure and according to the complexity of the surgical procedure, when there are no administrative or social reasons that prolong the stay.
	Unplanned return to operating theatre	Unplanned return to operating theatre within hospital stay or for a complication occurring directly or indirectly from the index surgical procedure.
	Unplanned admission to the Intensive Care Unit or Intermediate Medical Care Unit	Any unplanned admission to the Intensive Care Unit or Intermediate Medical Care Unit required for a complication occurring directly or indirectly from the surgical procedure.
	Unplanned readmission to hospital	First admission back to hospital related to the primary stay within 30 days of surgery.
**Important outcomes**	Pain	Level of pain at rest reported by the patient, using a validated scale.
	Surgical site infection	Infection related to a surgical procedure that occurs at skin and subcutaneous tissue of the incision (superficial incisional), at deep soft tissues of incision (deep incisional) or at part of the body deeper than the fascial/muscle layers that is opened or manipulated during the surgical procedure (organ/space surgical) within 30 days of the procedure, or within 90 days if prosthetic material is implanted at surgery.

		Includes any of the following: superficial incisional surgical site infection, deep incisional surgical site infection, organ/space surgical site infection.
	Postoperative nausea and vomit	Nausea and/or vomiting experienced by a patient following surgery, with its severity properly assessed by validated tools.
	Postoperative delirium	Delirium occurring in patients who have undergone a surgical procedure and anaesthesia during the in-hospital postoperative period.
	Postoperative major complications	Measure to aggregate relevant surgical complications using validated tools, which may include the following:<br>1. Death: Patient who died within 30 and 90 days after surgery.<br>2. New myocardial infarction.<br>3. Newly developed or significantly worsened congestive heart failure.<br>4. Persistent ventricular tachyarrhythmia.<br>5. New complete A-V block.<br>6. Severe hypotension (including cardiac arrest).<br>7. Pulmonary embolism.<br>8. Respiratory failure.<br>9. New cerebral hypoxia/ thrombosis/intracranial haemorrhage.<br>10. Renal failure.
	Unforeseen postoperative disability or harm	Permanent or temporary disability or harm that was likely initiated as a consequence of unexpected situations caused by perioperative complications.
	Postoperative quality of recovery	Quality of recovery reported by patients who have undergone a surgical procedure, using validated scores.

Core Process Measures Set for Patient Safety in Perioperative Care

Category	Measure	Definition
**Critical and feasible process measures**	Performing of venous thromboembolism prophylaxis	Patients undergoing high-risk orthopaedic or abdominal surgery are prescribed venous thromboembolism prophylaxis perioperatively.

	Labelling of surgical pathology specimens	Surgical pathology specimens are labelled according to recommendations, including labelled, filled containers, correct laterality, correct tissue type, patient name, and correct patient name.
**Important and feasible process measures**	Performing of perioperative glucose monitoring in diabetic patients	A perioperative glucose monitoring is conducted in diabetic patients by a knowledgeable and trained professional based on the best available evidence.
	Marking of surgery side and stoma site	The surgery side and stoma site are marked if indicated.
	Performing of high mortality surgeries under the direct supervision of a consultant surgeon and anaesthesiologist	Surgical procedures with predicted mortality >10% are conducted under the direct supervision of a consultant surgeon and anaesthesiologist.
	Compliance of post-anaesthesia medical records standards	Post-anaesthesia medical records are compliant with local recommendations. The following are recorded: information about patient evaluation on admission and discharge from Post-Anaesthesia Care Unit or admission to the Intensive Care Unit, a time-based record of vital signs and level of consciousness, time-based record of drugs administered, dosage and route of administration, type and counts of intravenous fluids administered, including blood and blood products, and post-anaesthesia visits.
	Performing of venous thromboembolism and bleeding risk assessment	Assessment of venous thromboembolism and bleeding risk using risk assessment criteria is conducted on admission and within 24 hours of admission.
	Administration of appropriate antibiotic prophylaxis	An appropriate antibiotic is given as per local guidelines.
	Application of Surgical Safety Checklist	A surgical Safety Checklist, namely WHO Surgical Safety Checklist, is applied.
	Compliance of medical records standards	Medical records are compliant with local recommendations. Information about discharge needs assessment and venous thromboembolism prophylaxis is recorded.
	Performing of risk assessment for pressure ulcers	Risk assessment for pressure ulcers using a standardised scale upon admission is conducted and appropriate measures will be put in place in each case.

	Recording of analgesic supplementation	The analgesic supplementation by any route at 24 hours is recorded.
	Recording of length of stay in Post-Anaesthesia Recovery Area	The length of stay in Post-Anaesthesia Recovery Area stay is recorded.
	Performing of preoperative preassessment	Preassessment according to local recommendations is conducted sufficient time before the day of surgery.
	Measurement of intraoperative blood loss	Intraoperative blood loss is measured and recorded.
	Use of early warning systems	Early warning systems are used at ward care.
	Recording of discharge destination	The discharge destination is recorded if the patient wishes to inform the staff.
	Assessment of pain	Whether pain is measured within the postoperative period, including predischarge and after discharge.

Core Structure Measures Set for Patient Safety in Perioperative Care

Category	Measure	Definition
**Critical and feasible structure measures**	Availability of perioperative up-to-date medication list	A preoperative and discharge up-to-date medication list is available in the clinical records.
	Availability of equipment to administer oxygen	Equipment to administer oxygen to all patients undergoing procedures under sedation by anaesthesiologists is available.
	Availability of specialised equipment for the management of difficult airways	Specialised equipment for the management of difficult airways is available where anaesthesia is given.
	Availability of defibrillators with cardiac pacing mode	Defibrillators with cardiac pacing mode are available.

	Availability of equipment for fluid and blood warming and rapid transfusion	Equipment for fluid and blood warming and rapid transfusion is available.
	Existence and dissemination of protocol for major haemorrhage	There is a well-defined internal protocol for major haemorrhage defined, including clinical laboratory and logistic responses, that is disseminated among professionals.
	Existence and dissemination of resuscitation policy	There is an internal policy for resuscitation defined and disseminated among professionals.
	Existence and dissemination of sedation policy	There is a well-defined internal policy for sedation that includes the training required by the sedation provider, all subspecialty areas and facility specifications, and this policy is disseminated among professionals.
	Existence and dissemination of emergency drugs policy	There is a well-defined internal policy that ensures emergency drugs are available where anaesthesia is given and adequately stored defined and this policy is disseminated among professionals.
	Existence and dissemination of management of anaesthesiologist procedures' complications policy	There is a well-defined internal policy for the management of complications of anaesthesiologist procedures and this policy is disseminated among professionals.
	Existence and dissemination of critical care referral policy	There is an internal policy for critical care referral defined and disseminated among professionals.
	Existence and dissemination of end-of-life care policy	There is an internal policy for end-of-life care defined and disseminated among professionals.
**Important and feasible structure measures**	Existence and dissemination of preoperative preparation policy	There is an internal policy for preoperative preparation defined and disseminated among professionals, including the following: fasting, blood typing, thromboprophylaxis, preoperative diabetes management, anaemia treatment in potential bleeding patients, and allergies.
	Availability of properly designed transfer trolleys	Properly designed transfer trolleys meet the requirements of the following list: oxygen cylinders, masks, tubing, infusion poles, equipment to secure and support airway and assist ventilation, provision of clamps for drainage tubes, protective sides, head down tilt possible are available.
	Standard compliant anaesthesia equipment	Equipment used to provide anaesthesia, including monitoring equipment, complies with existing local recommendations.

	Existence and dissemination of criteria for discharge policy	Internal criteria for discharge from recovery ward are defined and disseminated among professionals.
	Existence and dissemination of management of morbidly obese patients' policy	There is an internal policy for managing morbidly obese patients defined and disseminated among professionals.
	Existence and dissemination of remote site anaesthesia policy	There is an internal policy for remote site anaesthesia defined and disseminated among professionals.
	Availability of devices for maintaining or raising the patient's temperature	Devices for maintaining or raising the patient's temperature are available, including control of theatre temperature.
	Availability of blood storage facilities in close proximity to emergency theatres	Blood storage facilities are in close proximity to emergency theatres and contain 0 rhesus-negative blood.
	Availability of adequate Post-Anaesthesia Care Unit equipment	The Post-Anaesthesia Care Unit equipment includes:<br>- At bedside: pulse oximetry, ECG, and non-invasive blood pressure monitoring<br>- Immediately available: capnograph, 12 lead ECG, nerve stimulator, thermometer.
	Existence of an internal system for ordering, storing, recording and auditing controlled drugs	There is an internal system for ordering, storing, recording and auditing controlled drugs (e.g. morphine, fentanyl) in all postoperative areas where they are used.
	Existence and dissemination of staff training policy	There is an internal policy for staff training for both technical and non-technical skills defined, including in resuscitation, and disseminated among professionals.
	Existence and dissemination of patients' handover of care policy	There is an internal policy for the handover of care of the patient from one team to the other throughout the perioperative pathway defined and disseminated among professionals.
	Existence and dissemination of management and reporting of adverse events and near miss events policy	There is an internal policy for the management and reporting of adverse events and near miss events relating to the perioperative period defined and disseminated among professionals.
	Availability of consultant anaesthesiologist leading the	A consultant anaesthesiologist is responsible for leading the anaesthetic preoperative assessment service.

	anaesthetic preoperative assessment service	
	Existence and dissemination of referral pathways policy	There is a set of established guidelines within the organisation for referring patients to other medical specialties in order to streamline the diagnostic process.
	Existence and dissemination of flagging abnormal results policy	There is an internal policy to ensure that abnormal results of investigations are flagged to the relevant person in a timely manner defined and disseminated among professionals.
	Existence and dissemination of removing endotracheal tubes and supraglottic airways policy	There is an internal procedure for removing endotracheal tubes and supraglottic airways defined and disseminated among professionals.
	Availability of alternative language leaflets or videos and interpreters	Alternative language leaflets, other augmentative forms of communication, or videos and interpreters appropriate to the needs of the local population are available to patients and caregivers.
	Existence and dissemination of internal policy for senior clinicians on defined limits of care and resuscitation	There is an internal policy for senior clinicians to discuss the defined limits of care and resuscitation that is disseminated among professionals.
	Existence and dissemination of maintenance and replacement programme policy for anaesthetic equipment	There is a defined internal policy for planned maintenance and replacement programme for anaesthetic equipment defined, including naming a consultant to oversee the provision of anaesthetic equipment that is disseminated among professionals.
	Adequate number of surgical theatres	The number of existing theatres (excluding radiology suites, dedicated obstetric, minor operations but including day theatre) is considered adequate according to national recommendations.
	Existence and dissemination of clinical team patients visit policy	There is an internal policy for a member of the anaesthetic/clinical team to visit patients within 24 hours following the surgery defined and disseminated among professionals.
	Facilities for rest for on-call/on-duty staff are available	Facilities for rest for on-call/on-duty staff are available.
	Existence and dissemination of internal policy for receiving patient and caregiver feedback, including complaints	There is an internal policy for receiving feedback from patients and caregivers, including complaints, in place.
