# Supplementary material for: Core Measure Set for Patient Safety in Perioperative Care: A Clinical Practice-Oriented Consensus Study
Source: Int J Public Health. 2026 Mar 2;71:1609159. doi: 10.3389/ijph.2026.1609159 (PMC12989447; doi:10.3389/ijph.2026.1609159)
Supplement: Supplementary file 3 [file DataSheet3.pdf]

International Journal of Public Health

Title: **Core Measure Set for Patient Safety in Perioperative Care: A Clinical Practice-Oriented Consensus Study**

Supplementary file S3 - List of measures prioritised during the consensus process, including summary statistics from rounds 1 and 2 of the Modified eDelphi Technique, along with the scores for the discussed measures at the CMS Consensus Conference (European Union, 2023)

	**Round 1**						**Round 2**						**CMS Consensus Conference**
	**Importance**			**Feasibility**			**Importance**			**Feasibility**			
**Measure name**	7-9 % agree ment	Mean	SD	7-9% agree ment	Mean	SD	7-9% agree ment	Mean	SD	7-9% agree ment	Mean	SD	Score
Availability of Written or Audiovisual Patient Education Material*							72.50%	6.93	±1.16	76.92%	7.31	±1.56	NA
Facilities for privacy and confidentiality during preoperative discussion and examination are provided	63.64%	6.65	±1.89	68.52%	6.78	±1.97	71.43%	6.71	±1.59	69.39%	6.69	±1.91	NA
A consultant anaesthesiologist is responsible for leading the anaesthetic preoperative assessment service	76.36%	7.49	±1.34	81.48%	7.33	±1.54	83.67%	7.49	±1.19	87.76%	7.45	±1.46	NA
There is an internal policy for preoperative preparation defined and diffused among professionals, including all the following: fasting, investigations, blood typing, thromboprophylaxis, perioperative diabetes management, and allergies	92.59%	7.91	±1.03	83.33%	7.31	±1.46	97.96%	7.98	±0.92	87.76%	7.18	±1.37	NA
There is an agreed internal policy for referral pathways to other specialties to expedite further investigations that is diffused among professionals	80.00%	7.11	±1.14	71.70%	6.91	±1.64	85.71%	6.98	±0.95	75.00%	6.79	±1.53	NA
There is a defined internal policy to ensure that abnormal	92.73%	7.58	±1.06	72.22%	6.58	±1.76	93.88%	7.53	±1.01	75.51%	6.80	±1.69	NA

results of investigations are flagged to the relevant person in a timely manner defined that is diffused among professionals													
There is a defined internal policy to give information to patients and caregivers about the possible side effects of pain relief drugs that is diffused among professionals	78.18%	7.33	±1.33	64.15%	6.66	±1.60	85.71%	7.27	±1.21	69.39%	6.65	±1.52	9
There is an internal policy that determines the grade, experience and competency-based training of the nurse undertaking preoperative assessments and accompanying the patient to the operating department defined and diffused among professionals, particularly nurse managers	69.09%	6.89	±1.29	71.15%	6.77	±1.57	71.43%	6.84	±1.15	68.75%	6.50	1.59	NA
Preassessment according to local recommendations is conducted at least the day before surgery	88.89%	7.69	±1.32	79.63%	7.22	±1.51							NA
An anaesthetic risk assessment informing the process of consent is performed. The patient is informed of the risk	87.04%	7.87	±1.31	68.52%	7.17	±1.40				66.67%	7.13	±1.35	NA
The patient is told not to remove hair from the surgical site and to have a preoperative shower at home	60.38%	6.60	±1.68	59.26%	6.30	±1.69	60.42%	6.52	±1.42	64.58%	6.33	±1.60	NA
A preoperative up to date medication list is available in the clinical records	98.11%	8.23	±0.87	71.70%	7.17	±1.47	97.92%	8.35	±0.85	75.00%	7.23	±1.46	NA
A preoperative glucose monitoring is conducted in diabetic patients by a knowledgeable and trained	84.91%	7.47	±1.14	85.19%	7.61	±1.20	87.50%	7.83	±1.23	87.50%	7.60	±1.08	NA

professional based on the best available evidence													
Re-assess of venous thromboembolism and bleeding risk using risk assessment criteria is conducted on admission and within 24 hours of admission	90.57%	7.74	±1.01	74.07%	7.09	±1.33				75.00%	7.00	±1.15	NA
Verbal and written information on venous thromboembolism prevention is offered to the patient	66.04%	6.98	±1.40	69.23%	6.87	±1.33	77.08%	7.00	±1.38	68.09%	6.77	±1.20	4
Patients assessed to be at risk of venous thromboembolism are offered prophylaxis in accordance with best practices	94.23%	7.81	±0.92	69.23%	7.02	±1.17				80.85%	7.09	±1.02	NA
A preoperative chlorhexidine shower is taken or given to the patient	46.15%	5.50	±2.32	52.83%	6.09	±1.90	47.92%	5.38	±2,28	54.17%	6.08	±1.72	NA
The stoma site is marked if indicated	74.51%	7.22	±1.53	69.81%	6.87	±1.65	83.33%	7.54	±1.47	75.00%	6.96	±1.68	NA
Preoperative assessment by a stoma care nurse is conducted if indicated	60.00%	6.60	1.56	63.46%	6.40	1.44	66.67%	6.94	±1.38	70.21%	6.47	±1.43	NA
Risk assessment for pressure ulcers using a standardized scale upon admission is conducted	69.57%	6.76	±1.23	76.60%	7.11	±1.39	77.08%	7.00	±1.08				NA
The cancellation rate is measured and is detailed by cause	61.70%	6.28	±1.47	63.04%	6.48	±1.71	78.72%	6.66	±1.29	69.57%	6.59	±1.45	9
Patient Satisfaction with Perisurgical Education*							52.63%	6.26	±1.61	33.33%	6.08	±1.16	NA
Time from referral to consultation	61.11%	6.30	±1.72	58.82%	6.43	±2.04	77.08%	6.60	±1.42	68.75%	6.81	±1.74	7
Time from diagnosis to surgery	72.22%	6.50	±1.79	67.31%	6.67	±1.71	81.25%	6.83	±1.48	70.83%	6.81	±1.59	3
Proportion of prospective surgical patients that are the target of a cardiovascular risks' discussion preoperatively	64.81%	6.44	±1.56	36.04%	5.77	±1.90	83.33%	6.85	±0.99	31.25%	5.88	±1.61	7

Proportion of prospective surgical patients that undergo a cardiovascular risks' assessment preoperatively	87.04%	7.17	±1.07	51.92%	6.27	±1.70	91.67%	7.29	±1.01	56.25%	6.52	±1.21	8
Proportion of prospective surgical patients that undergo a measurement of vital signs as well as a cardiac physical examination preoperatively	79.63%	7.02	±1.30	63.46%	6.54	±1.60	87.50%	7.17	±1.01	72.34%	6.79	±1.33	8
Proportion of prospective surgical patients that undergo echocardiography assessment preoperatively	57.41%	6.26	±1.68	64.00%	6.08	±1.40	62.50%	6.48	±1.53	77.08%	7.19	±1.18	NA
Proportion of prospective surgical patients that undergo electrocardiographic (ECG) assessment preoperatively	68.52%	6.56	±1.73	70.59%	6.94	±1.46	79.17%	7.04	±1.42	81.25%	7.35	±1.20	NA
Proportion of prospective surgical patients that have their full blood count, coagulation profile and renal function checked preoperatively	75.93%	7.07	±1.61	78.85%	7.21	±1.46	83.33%	7.10	±1.49	82.98%	7.38	±1.44	NA
Proportion of prospective surgical patients that undergo functional status evaluation preoperatively	86.79%	7.17	±0.99	59.62%	6.44	±1.32	89.58%	7.15	±0.94	68.09%	6.68	±1.12	4
Proportion of prospective surgical patients that undergo frailty assessment preoperatively	82.69%	7.04	1.04	54.00%	6.20	±1.59	87.50%	6.94	±1.06	58.70%	6.48	±1.28	NA
Cardiac evaluation before hospital discharge	71.43%	6.47	±1.67	60.42%	6.42	±1.58	76.60%	6.51	±1.57	64.44%	6.67	±1.18	2
The discharge destination from the hospital (e.g. patient's previous home or residence, short-term rehabilitation facility, or long-term care facility) is known by the healthcare professional and is adequate to the status of the patient	63.83%	6.62	±1.46	74.47%	6.60	±1.31	73.33%	7.00	±1.21	84.44%	6.96	±1.14	NA

Properly designed transfer trolleys meet the requirements of the following list: oxygen cylinders, masks, tubing, infusion poles, equipment to secure and support airway and assist ventilation, provision of clamps for drainage tubes, protective sides, head down tilt possible are available	86.54%	7.65	±1.25	76.92%	7.15	±1.73	91.67%	7.67	±1.30	81.25%	7.29	±1.68	NA
Equipment to administer oxygen to all patients undergoing procedures under sedation by anaesthesiologists is available	94.23%	8.27	±0.94	84.62%	7.62	±1.49	95.83%	8.40	±0.80	85.42%	7.60	±1.43	NA
Specialised equipment for the management of difficult airways is available where anaesthesia is given	96.08%	8.24	±0.92	88.46%	7.56	±1.63							NA
There is a well-defined internal policy for sedation that includes the training required by the sedation provider, all subspecialty areas and facility specifications and this policy is diffused among professionals	90.20%	7.65	±1.16	82.35%	7.43	±1.76	93.75%	7.98	±0.98	87.50%	7.54	±1.70	NA
There is a well-defined internal policy that ensures emergency drugs are available where anaesthesia is given and adequately stored defined and this policy is diffused among professionals	94.12%	7.90	±1.02	82.35%	7.47	±1.64	95.83%	8.13	±0.90	85.42%	7.58	±1.58	NA
There is a well-defined internal policy for the management of complications of anaesthesiologist procedures, and this policy is diffused among professionals	90.20%	7.71	±1.17	80.39%	7.24	±1.59	93.75%	8.02	±0.97	85.42%	7.42	±1.43	NA
Devices for maintaining or raising the patient's temperature are available,	86.54%	7.62	±1.16	88.24%	7.53	±1.65							NA

including control of theatre temperature													
Defibrillators with cardiac pacing mode are available	93.75%	8.10	±0.95	91.84%	7.76	±1,16							NA
Equipment for fluid and blood warming and rapid transfusion is available	86.96%	7.70	±1.19	89,58%	7.67	±1.40							NA
Blood storage facilities are in close proximity to emergency theatres and contain 0 rhesus negative blood	89.13%	7.48	±1.19	78.26%	7.11	±1.55							NA
There is a well-defined internal protocol for major haemorrhage defined, including clinical laboratory and logistic responses, that is diffused among professionals	89.13%	7.72	±1.13	86.96%	7.52	±1.54	95.65%	8.24	±0.79	93.62%	7.83	±1.39	NA
At least one member of recovery staff certified as an advanced life support provider or equivalent is available at any given time. An additional certified in advanced life support staff is available	92.86%	7.98	±1.01	69.05%	6.90	±1.54							NA
An appropriate antibiotic is given as per local guidelines	88.46%	7.77	±1.02	88.68%	7.36	±1.28							NA
Equipment used to provide anaesthesia, including monitoring equipment, complies with existing local recommendations	88.24%	7.61	±1.10	79.25%	7.19	±1.13							NA
Surgical procedures with predicted mortality >10% are conducted under the direct supervision of a consultant surgeon and anaesthesiologist	86.54%	7.46	±1.19	69.81%	6.96	±1.24	89.58%	7.71	±1.18	81.25%	7.13	±1.05	NA
The WHO Surgical Safety Checklist is applied	90.38%	7.65	±1.05	75.47%	7.17	±1.47							NA
Appropriate hair removal (using clippers, not a razor) is	67.31%	6.48	±1.69	56.60%	6.42	1.47	77.08%	6.60	±1.49	64.58%	6.52	±1.30	6

performed on patients, when warranted													
Intraoperative blood loss is measured and recorded	86.54%	7.38	±1.05	78.85%	7.10	±1.44							NA
Surgical pathology specimens are labelled according to recommendations, including labelled, filled containers, correct laterality, correct tissue type, patient name, and correct patient name	94.23%	7.90	±0.89	80.77%	7.33	±1.29							NA
The turnover time between cases is measured	62.75%	6.49	±1.95	73.08%	7.12	±1.82	77.08%	6.67	±1.65	81.25%	7.38	±1.63	NA
The operating time is recorded	78.00%	7.16	±1.70	94.12%	7.94	±1.02							NA
After general or regional anaesthesia or sedation, patients recover in a designated area, compliant with the existing recommendations¤													NA
Failed regional anaesthesia block	62.75%	6.25	±1.60	43.75%	5.50	±1.90	68.75%	6.60	±1.29	45.83%	5.96	±1.60	NA
Incomplete regional anaesthesia block¶	64.00%	6.16	±1.56	33.33%	5.25	±1.93							NA
Inadequate regional block¶	62.00%	6.12	±1.54	33.33%	5.31	±1.90	73.17%	6.46	±1.57	46.51%	5.81	±1.73	NA
Unintended epidural vessel penetration	75.51%	6.61	±1.71	37.50%	5.23	±1.98				41.67%	5.85	±1.61	NA
Failed attempt of endotracheal intubation	80.00%	6.92	±1.43	66.67%	6.46	±1.40	89.58%	7.29	±1.23	77.08%	6.85	±1.23	NA
Mucosal trauma after intubation	70.00%	6.50	±1.44	39.58%	5.60	±1.63	70.83%	6.83	±1.27	45.83%	6.08	±1.54	NA
Intraoperative airway complications	90.00%	7.26	±1.23	58.33%	6.40	±1.52				58.33%	6.69	±1.39	NA
Severe hypotension	89.80%	7.41	±1.14	59.18%	6.63	±1.46				62.50%	6.81	±1.63	NA
Wrong site surgery	94.12%	8.14	±0.86	85.71%	7.65	±1.29							NA
Intraoperative complications¤	93.88%	8.02	±0.90	67.39%	7.11	±1.47							NA
Intraoperative blood transfusion	89.58%	7.48	±1.01	84.78%	7.83	±1.30							NA
Unanticipated transfusion of any blood products	88.89%	7.58	±1.02	67.44%	7.14	±1.74				76.09%	7.35	±1.74	NA

Length of surgery	82.22%	7.11	±1.48	83.72%	7.88	±1.28							NA
Surgical team satisfaction	56.10%	6.56	±1.60	38.10%	5.74	±2.12	56.25%	6.75	±1.40	36.96%	6.09	±1.87	NA
The Post-Anaesthesia Care Unit equipment includes:<br>- At bedside: pulse oximetry, ECG and Non-invasive Blood Pressure Monitoring<br>- Immediately available: capnograph, 12 lead ECG, nerve stimulator, thermometer	92.16%	7.80	±1.18	90.20%	7.53	±1.26							NA
There is an internal system for ordering, storing, recording and auditing controlled drugs (e.g. morphine, fentanyl) in all postoperative areas where they are used	84.31%	7.31	±1,32	82,35%	7.22	±1.36							NA
There is an internal procedure defined for removing endotracheal tubes and supraglottic airways and that is diffused among professionals	73.47%	6.51	±1.72	70.59%	6.76	±1.55	79.17%	6.77	±1.53	75.00%	6.83	±1.56	NA
There is an internal policy for a member of the anaesthetic/clinical team to visit patients within 24 hours following the surgery (ASA grade 3,4,5: epidural on ward, invasive monitoring in-situ or as requested by health care worker) defined.	78.43%	6.92	±1.28	73.08%	7.00	±1.29				75.00%	7.00	±1.31	NA
There is an internal policy defined to advise patients and caregivers on wounds and dressing care	76.92%	6.75	±1.37	63.46%	6.65	±1.40				60.42%	6.73	±1.44	NA
Internal criteria for discharge from recovery ward are defined and diffused among professionals	86.00%	7.26	±1.11	72.00%	6.92	±1.32	91.67%	7.42	±0.93	76.60%	7.09	±1.25	NA
There is an internal policy for reviewing the morbidity, mortality and all untoward	90.38%	7.62	±1.04	68.63%	6.90	±1.52				66.67%	6.81	±1.51	NA

incidents of all anaesthetic activity defined and documented													
Whenever critical care admission is not possible, an appropriate care package that is previously communicated to the patient is provided	80.77%	6.71	±1.58	37.50%	5.42	±1.71	83.33%	6.60	±1.71	41.67%	5.52	±1.63	NA
Whenever there is a lack of appropriate facilities elsewhere, critically ill patients are only held in recovery/PACU if level 3 critical care standard of treatment can be provided	84.31%	6.63	±1.47	44.68%	5.38	±1.90	85.42%	6.58	±1.48	56.25%	5.52	±1.75	NA
Whether safety to discharge from the surgical suite using a standardized scale is assessed	75.00%	6.27	±1.66	48.98%	5.90	±1.68				56.25%	6.04	±1.52	NA
Transfer from theatre to Post-Anaesthesia Care Unit is done by suitably trained staff under the supervision of an anaesthesiologist	84.31%	6.94	±1.31	55.32%	6.00	±1.79	91.67%	7.08	±1.05	62.50%	6.29	±1.52	NA
A complete and formal handover is performed with verbal and written instruction	86.27%	7.59	±1.19	55.32%	5.98	±1.79				58.33%	6.10	±1.64	NA
Patients with a mortality risk of>10% are admitted to critical care	76.47%	7.06	±1.62	56.25%	6.48	±1.74				66.67%	6.69	±1.63	NA
Eligibility to bypass the Post-Anaesthesia Care Unit using a standardized scale is assessed	70.59%	6.16	±1.80	54.17%	5.75	±1.75	79.17%	6.31	±1.51	68.75%	6.33	±1.36	NA
Patients have a postoperative multidisciplinary team consultation, and a discharge coordinator is present	58.82%	6.16	±1.85	41.67%	5.79	±1.59	66.67%	6.23	±1.65	43.75%	5.90	±1.48	NA
Patient’s supporter(s) are immediately post-surgery updated about the outcome of surgery by a member of the medical/nursing team	65.38%	6.69	±1.78	43.75%	6.02	±1.64	72.92%	6.90	±1.70	48.94%	6.17	±1.50	NA

The postoperative morphine consumption at 6, 24 and 48 hours is recorded	66.00%	6.56	±1.67	82.61%	7.09	±1.42	77.08%	6.83	±1.46				NA
Postoperative delirium screening is conducted in patients over 65 years old according to local recommendations	75.51%	6.78	±1.35	63.64%	6.59	±1.60				69.57%	6.67	±1.47	NA
The analgesic supplementation by any route at 24 hours is recorded	68.75%	6.75	±1.52	79.07%	7.09	±1.45	80.85%	7.06	±1.22				NA
Post-anaesthesia medical records are compliant with local recommendations. The following are recorded: Information about patient evaluation on admission and discharge from Post-Anaesthesia Care Unit or admission to the Intensive Care Unit, a time-based record of vital signs and level of consciousness, time-based record of drugs administered, dosage and route of administration, type and counts of intravenous fluids administered, including blood and blood products, and post-anaesthesia visits	84.78%	7.26	±1.34	60.98%	6.41	±1.64	93.75%	7.50	±1.04	75.56%	6.69	±1.39	NA
The patient's condition is continuously monitored in Post-Anaesthesia Care Unit	88.89%	7.49	±1.18	60.98%	6.51	±1.69				71.11%	6.78	±1.63	NA
Supplemental oxygen postoperatively is given to all patients	52.17%	5.30	±2.59	56.10%	6.37	±1.82	58.70%	5.59	±2.31	62.22%	6.56	±1.74	NA
Pain is measured whenever each set of vital signs is measured, or it is measured to a specified regularity (in case vital signs are being continuously monitored)	67.39%	6.93	±1.67	70.73%	6.80	±1.40	76.09%	7.17	±1.51	71.11%	6.76	±1.70	NA

Pain is brought under control within 48 hours postoperatively	71.11%	6.69	±1.66	55.00%	6.15	±1.69	80.43%	6.91	±1.56	66.67%	6.51	±1.57	NA
Antiemetic treatment is administered when nausea and vomiting are present during acute pain	77.78%	6.84	±1.36	53.85%	6.33	±1.62				71.11%	6.64	±1.67	NA
Phlebitis screening using score is conducted daily	64.44%	6.33	±1.36	48.72%	6.26	±1.82	69.57%	6.46	±1.32	57.78%	6.51	±1.57	NA
Early postoperative mobilization is performed	88.64%	7.55	±1.05	56.41%	6.21	±1.46				68.89%	6.64	±1.11	NA
There is a record kept of the number of patients who received antibacterials beyond the recommended postoperative period	65.12%	6.63	±1.39	52.63%	6.29	±1.74	76.09%	6.70	±1.25	65.91%	6.55	±1.47	NA
The Post-Anaesthesia Care Unit's length of stay is recorded	68.18%	6.91	±1.37	82.05%	7.10	±1.47	79.55%	7.11	±1.28	86.36%	7.27	±1.40	NA
Early warning systems are used at ward care	88.64%	7.64	±1.14	65.79%	6.89	±1.58				75.00%	7.07	±1.49	NA
Clear information on discharge is provided to the patient and carers, including how to contact healthcare professionals for advice and support following discharge	83.72%	7.49	±1.16	46.15%	6.18	±1.73				59.09%	6.52	±1.60	NA
The discharge destination is recorded	79.07%	7.21	±1.22	73.68%	6.97	±1.46				81.82%	7.05	±1.34	NA
Medical records are compliant with local recommendations. Information about discharge needs assessment and venous thromboembolism prophylaxis is recorded	86.36%	7.52	±1.28	58.97%	6.38	±1.69				79.55%	6.77	±1.45	NA
Arrangements are in place for the multidisciplinary management of elderly patients, including routine daily input from internal medicine or geriatric specialists	79.55%	7.18	±1.26	37.84%	5.62	±1.78				48.84%	6.28	±1.55	NA
Recovery area complies with local standards	86.05%	7.51	±1.11	64.86%	6.65	±1.58				81.40%	7.07	±1.30	NA

Recurrence or progression of the disease	66.00%	6.62	±1.81	36.96%	5.43	±1.75	79.17%	6.88	±1.74	35.42%	5.60	±1.57	NA
Time to recurrence or progression of disease	64.00%	6.32	±1.84	32.61%	5.54	±1.82	75.00%	6.52	±1.76	29.17%	5.67	±1.64	NA
Long-term ‘disease-specific’ survival	70.00%	6.46	±1.82	34.78%	5.52	±1.76	77.08%	6.71	±1.63	35.42%	5.67	±1.54	NA
Overall long-term survival	70.00%	6.54	±1.80	52.17%	6.13	±1.92	77.08%	6.85	±1.44	66.67%	6.35	±1.73	3
Cure of underlying disease	64.58%	6.48	±1.89	39.13%	5.43	±1.90	70.83%	6.83	±1.53	51.06%	5.79	±1.66	NA
Mortality	87.76%	7.51	±1.47	76.09%	7.15	±1.70							NA
Postoperative awareness	71.43%	6.76	±1.21	36.96%	5.52	±1.80	81.25%	6.85	±1.04	33.33%	5.63	±1.50	Not scored
Short-term level of functional dependence	70.00%	6.60	±1.34	34.78%	5.65	±1.55	77.08%	6.65	±1.28	37.50%	5.69	±1.46	NA
Long-term level of functional dependence	70.00%	6.74	±1.46	28.26%	5.59	±1.57	78.72%	6.77	±1.31	25.00%	5.56	±1.31	NA
Functional status	75.51%	6.84	±1.43	43.18%	6.16	±1.28				47.92%	6.29	±0.97	NA
Time to resume to normal level of mobility	63.83%	6.49	±1.58	37.33%	5.74	±1.76	70.21%	6.43	±1.41	29.17%	5.83	±1.49	NA
Time to resume work and other usual life roles	63.04%	6.43	±1.65	32.56%	5.84	±1.71	72.34%	6.49	±1.42	27.66%	5.85	±1.42	NA
Postoperative sleep quality	55.32%	6.26	±1.55	20.93%	5.30	±1.64	61.70%	6.15	1.52	14.89%	5.36	±1.47	NA
Physical recovery	71.11%	6.76	±1.33	24.39%	5.32	±1.72	82.98%	6.89	±1.09	23.91%	5.52	±1.44	9
Postoperative neuropsychologic performance deterioration	67.39%	6.59	±1.26	23.81%	5.29	±1.83	74.47%	6.53	±1.20	19.57%	5.37	±1.63	NA
Time to gastro-intestinal recovery	65.22%	6.76	±1.31	35.71%	5.79	±1.80	76.60%	6.68	±1.16	30.43%	5.87	±1.44	NA
Time to mobilization	71.74%	7.11	±1.22	54.76%	6.45	±1.77	78.72%	6.94	±1.15	65.22%	6.54	±1.43	8
Physiotherapy tolerance	50.00%	6.28	±1.40	30.95%	5.50	±1.74	57.45%	6.21	±1.23	40.00%	5.91	±1.42	NA
Permanent disability or harm	91.30%	7.78	±1.21	41.46%	5.90	±1.91				46.67%	6.09	±1.69	NA
Cognitive recovery	76.09%	7.11	±1.18	21.95%	5.29	±1.83				8.89%	5.31	±1.35	NA
Psychological recovery	67.39%	6.52	±1.46	19.05%	4.93	±1.84	74.47%	6.36	±1.37	13.33%	5.02	±1.55	NA
Overall recovery	80.00%	7.02	±1.66	26.19%	5.21	±1.93				17.78%	5.31	±1.66	NA
Postoperative quality of recovery	69.57%	6.74	±1.53	21.43%	5.26	±1.96	76.60%	6.72	±1.35	15.56%	5.38	±1.76	NA
Long-term impact on relatives and/or caregivers	56.52%	6.33	±1.68	14.63%	4.54	±2.10	59.57%	6.28	±1.41	11.11%	4.64	±1.91	NA
Fever	89.13%	7.57	±1.14	73.17%	7.27	±1.81				83.72%	7.74	±1.59	NA
Postoperative sepsis	97.83%	8.07	±1.00	60.98%	6.66	±1.65				69.77%	6.72	±1.32	NA
Septic Shock	95.65%	8.09	±1.06	70.73%	6.83	±1.69				81.40%	7.09	±1.38	NA

Postoperative Systemic inflammatory response syndrome	93.33%	7.84	±1.18	53.66%	6.46	±1.75				54.76%	6.52	±1.44	NA
Postoperative pneumonia	100.00%	7.98	±0.81	70.73%	6.93	±1.61				76.19%	7.00	±1.25	NA
Ventilator associated pneumonia	97.83%	7.93	±0.81	67.50%	6.83	±1.62				71.43%	6.83	±1.16	NA
Postoperative urinary tract infection	95.65%	7.83	±0.84	65.00%	6.83	±1.57				73.81%	6.98	±1.19	NA
Catheter-related bloodstream infections	93.48%	7.85	±0.95	63.41%	6.61	±1.79				73.81%	6.76	±1.34	NA
Postoperative Clostridium difficile infection / Colitis	95.65%	7.76	±0.90	60.98%	6.63	±1.69				69.05%	6.83	±1.38	NA
Postoperative endometritis	77.78%	6.96	±1.21	48.78%	6.17	±1.70				57.14%	6.26	±1.36	NA
Postoperative neurological Infection	91.11%	7.56	±0.99	58.54%	6.59	±1.68				64.29%	6.64	±1.26	NA
Surgical site infection¶	97.83%	7.98	±0.87	65.85%	7.00	±1.71	97.56%	7.93	±0.86	70.59%	6.79	±1.54	8
Superficial incisional surgical Site Infection¶	86.36%	7.57	±0.94	68.29%	6.90	±1.62							NA
Deep incisional surgical site Infection¶	95.65%	7.93	±0.87	60.98%	6.76	±1.67							NA
Organ/Space surgical site infection¶	93.48%	7.87	±0.90	63.41%	6.78	±1.65							NA
Antibiotics use	84.78%	7.37	±1.01	72.50%	7.20	±1.68				80.95%	7.50	±1.49	NA
Wound healing time	73.33%	6.64	±1.24	42.50%	6.00	±1.83	76.60%	6.51	±1.55	40.48%	6.05	±1.53	5
Time to orientation	65.22%	6.50	±1.29	34.15%	5.90	±2.00	70.21%	6.36	±1.38	30.95%	6.00	±1.72	NA
Unplanned return to operating theatre	93.48%	7.76	±1.03	73.17%	7.20	±1.79				83.33%	7.57	±1.48	NA
Readmission to the Intensive Care Unit or Intermediate Medical Care Unit	100.00%	7.98	±0.84	77.50%	7.33	±1.70							NA
Unplanned attendance to healthcare facility	93.48%	7.54	±0.85	52.50%	6.60	±1.85	95.74%	7.55	±0.75	52.38%	6.69	±1.47	NA
Unplanned readmission to hospital	97.78%	7.82	±0.87	70.00%	7.13	±1.87				80.49%	7.46	±1.54	NA
Length of stay	82.61%	7.33	±1.01	82.50%	7.53	±1.56							NA
Length of stay in Post-Anaesthesia Recovery Area	71.74%	6.89	±1.15	75.00%	7.40	±1.64	76.60%	6.70	±1.28				NA
Length of stay in the Intensive Care Unit	80.43%	7.30	±1.01	85.00%	7.55	±1.54							NA
Postoperative acute lung injury	95.65%	7.70	±0.96	60.00%	6.78	±1.67				66.67%	6.88	±1.47	NA

Postoperative respiratory failure	97.83%	8.00	±0.91	65.00%	6.88	±1.84				71.43%	7.02	±1.71	NA
Postoperative pulmonary complications	100.00%	7.98	±0.78	62.50%	6.88	±1.65				69.05%	7.00	±1.29	NA
Postoperative myocardial infarction	100.00%	7.98	±0.78	60.00%	6.83	±1.72				66.67%	6.98	±1.47	NA
Postoperative stroke	100.00%	8.17	±0.84	60.00%	6.83	±1.76				66.67%	7.05	±1.52	NA
Postoperative atrial fibrillation	95.56%	7.69	±0.82	62.50%	6.93	±1.77				71.43%	7.29	±1.51	NA
Postoperative Major Acute Cardiac Event (MACE)	100.00%	8.02	±0.81	60.00%	6.95	±1.66				59.52%	7.00	±1.38	NA
Postoperative acute kidney injury	97.78%	7.80	±0.82	60.00%	6.95	±1.69				61.90%	7.00	±1.38	NA
Postoperative Major Adverse Kidney Events (MAKE)	100.00%	8.00	±0.84	60.00%	6.93	±1.70				61.90%	7.02	±1.34	NA
Postoperative central nervous system failure	97.78%	7.80	±0.79	58.97%	6.69	±1.89				59.52%	6.79	±1.56	NA
Postoperative hepatic failure	97.78%	7.82	±0.81	57.50%	6.88	±1.81				59.52%	7.17	±1.43	NA
Postoperative haematology failure	97.73%	7.75	±0.83	52.50%	6.48	±1.88				57.14%	6.81	±1.45	NA
Bleeding complications	97.83%	7.96	±1.01	65.00%	7.03	±1.69				69.05%	7.12	±1.52	NA
Postoperative deep vein thrombosis	97.83%	7.76	±0.81	57.50%	6.78	±1.62				64.29%	7.05	±1.35	NA
Postoperative pulmonary embolism	100.00%	8.11	±0.81	62.50%	6.88	±1.68				66.67%	7.07	±1.47	NA
Postoperative major complications	100.00%	8.16	0.8	62.50%	6.73	1.49				69.05%	6.83	±1.30	NA
Reoccurrence of neuromuscular blockade	84.78%	6.78	±1.08	51.28%	6.26	±1.51				52.38%	6.21	±1.23	NA
Early extubation	66.67%	6.13	±1.55	61.54%	6.26	±1.68	74.47%	5.98	±1.62	66.67%	6.10	±1.33	NA
Postoperative composite pharyngolaryngeal adverse events	71.74%	6.59	±1.22	48.72%	5.95	±1.72	80.85%	6.74	±1.08	57.14%	6.19	±1.35	9
Recovery room airway complications	84.78%	7.02	±1.09	56.41%	6.62	±1.57				59.52%	6.52	±1.28	NA
Postoperative delirium	84.78%	7.02	±1.09	50.00%	6.53	±1.75				47.62%	6.45	±1.43	NA
Postoperative shivering	56.52%	6.37	±1.35	51.28%	6.46	±1.66	61.70%	6.38	±1.05	52.38%	6.10	±1.40	NA
Pressure ulcers	78.26%	7.15	±1.25	53.85%	6.59	±1.62				61.90%	6.86	±1.36	NA
Post-discharge nausea and vomiting	60.87%	6.43	±1.52	51.28%	6.28	±1.84	74.47%	6.66	±1.00	54.76%	6.36	±1.60	NA

Postoperative nausea and vomit¶^	58.70%	6.39	±1.25	66.67%	6.64	±1.66							NA
Postoperative nausea and vomit severity¶^	60.87%	6.57	±1.23	51.28%	5.97	±1.74	82.86%	6.97	±0.93	50.00%	6.06	±1.48	4
Need for rescue antiemetics	67.39%	6.50	±1.26	70.27%	6.73	±1.60	72.34%	6.57	±0.98	75.61%	6.83	±1.21	NA
Postoperative fatigue	54.35%	6.26	±1.38	25.64%	5.26	±1.66	63.83%	6.47	±1.16	23.81%	5.33	±1.48	NA
Opioid-related side-effects	65.22%	6.59	±1.32	43.59%	5.87	±1.77	74.47%	6.70	±1.02	47.62%	6.05	±1.26	Not scored
Postoperative mental health symptoms	56.52%	6.41	±1.37	32.43%	5.49	±1.95	65.96%	6.60	±1.18	28.57%	5.52	±1.48	NA
Postoperative adverse events¤	86.67%	7.18	±1.06	44.74%	6.11	±1.68							NA
Postdural puncture headache	76.09%	6.78	±1.33	52.63%	6.47	±1.77				60.98%	6.54	±1.52	NA
Postoperative pain intensity	82.61%	7.02	±1.16	58.97%	6.64	±1.60				70.73%	6.93	±1.25	NA
Time to first analgesic request	58.70%	6.33	±1.29	64.10%	6.72	±1.78	70.21%	6.43	±1.15	75.61%	6.90	±1.38	NA
Time to lowest pain score	47.83%	6.24	±1.33	46.15%	6.41	±1.86	55.32%	6.21	±1.31	41.46%	6.51	±1.45	NA
Analgesic consumption	66.67%	6.73	±1.35	66.67%	6.90	±1.78	74.47%	6.57	±1.19	78.05%	7.27	±1.44	NA
Chronic pain	68.89%	6.78	±1.33	30.77%	5.62	±1.80	72.34%	6.43	±1.30	26.83%	5.73	±1.42	NA
Independence from opioids	62.22%	6.51	±1.41	33.33%	5.64	±1.70	70.21%	6.47	±1.30	36.59%	6.10	±1.47	NA
Anastomotic leakage	88.00%	7.42	±1.17	58.33%	6.33	±1.32				65.96%	6.47	±1.54	NA
Alternative language leaflets or videos and interpreters appropriate to the needs of the local population are available to patients and caregivers	68.09%	6.83	±1.49	68.09%	6.51	±1.83	75.00%	6.90	±1.23	79.17%	6.67	±1.61	NA
There is an internal policy for senior clinicians to discuss the defined limits of care and resuscitation that is diffused among professionals	76.60%	7.11	±1.25	71.74%	6.78	±1.62	89.58%	7.13	±1.12	77.08%	6.88	±1.54	NA
There is a defined internal policy for planned maintenance and replacement programme for anaesthetic equipment defined, including naming a consultant to oversee the provision of anaesthetic equipment that is diffused among professionals	76.60%	6.89	±1.43	69.57%	6.67	±1.96	85.42%	6.94	±1.28	75.00%	6.75	±1.79	NA
There is an internal policy for anaesthetic emergencies	93.48%	7.89	±1.15	71.74%	6.89	±1.77	97.92%	8.02	±0.98	77.08%	6.92	±1.59	NA

defined and diffused among professionals													
There is an internal policy for managing morbidly obese patients defined and diffused among professionals	85.11%	7.38	±1.22	74.47%	7.06	±1.63	95.83%	7.58	±0.90	81.25%	7.08	±1.48	NA
There is an internal policy for remote site anaesthesia defined and diffused among professionals	80.43%	6.85	±1.50	73.91%	6.78	±1.86	87.50%	7.21	±1.24	79.17%	6.81	±1.72	NA
There is an internal policy for critical care referral defined and diffused among professionals	86.96%	7.78	±1.21	76.09%	6.98	±1.86	95.83%	7.96	±0.97	79.17%	6.96	±1.72	NA
There is an internal policy for resuscitation defined and diffused among professionals	93.62%	8.11	±1.16	78.26%	7.13	±1.69	97.87%	8.28	±0.97	82.98%	7.21	±1.61	NA
There is an internal policy for end-of-life care defined and diffused among professionals	93.33%	7.89	±1.10	73.33%	7.02	±1.75	95.74%	7.79	±0.95	76.60%	7.11	±1.67	NA
There is an internal policy for staff training for both technical and non-technical skills defined, including in resuscitation	91.30%	7.85	±1.15	68.89%	6.82	1.86				76.60%	6.98	±1.74	NA
Supervisory consultants are freely available to all junior anaesthesiologists	91.11%	7.73	1.19	47.73%	6.07	±2.03	91.49%	7.49	±1.12	59.57%	6.49	±1.96	NA
A pharmacist is available to consult with physicians and nurses over non-critical issues	84.44%	7.18	±1.02	50.00%	6.23	±1.76				59.57%	6.60	±1.60	NA
There is an internal policy for the handover of care of the patient from one team to the other throughout the perioperative pathway	82.22%	7.51	±1.18	75.00%	7.00	±1.81							NA
There is an internal policy for the management and reporting of adverse events and near miss events relating to the perisurgical period	88.37%	7.74	±1.14	69.77%	7.02	±1.87				78.26%	7.22	±1.74	NA
Facilities for rest for on-call/on-duty staff are available	81.82%	7.43	±1.42	65.12%	6.81	±1.70				78.72%	7.06	±1.57	NA

The hospital is a tertiary referral centre for specialist services, including pain	61.36%	5.80	±2.09	72.09%	6.56	±1.99	74.47%	6.04	±1.85	80.85%	6.89	±1.68	NA
The hospital number of beds is adequate according to national recommendations	72.73%	6.80	±1.72	53.49%	6.21	±2.36	87.23%	6.96	±1.57	61.70%	6.47	±2.05	NA
The hospital case volume is adequate according to national recommendation	76.74%	7.05	±1.20	48.84%	6.02	±2.29	87.23%	7.11	±1.04	61.70%	6.47	±2.05	NA
The average case volume per surgeon is adequate according to national recommendations	79.55%	7.23	±1.22	46.51%	6.00	±2.31	91.49%	7.45	±0.96	57.45%	6.34	±2.17	NA
There is number of accredited healthcare professionals, including anaesthetists, surgeons and perioperative specialized nurses that is considered adequate according to national recommendations	88.64%	7.64	±1.12	51.16%	6.12	±2.29	93.62%	7.72	±1.07	68.09%	6.53	±2.14	NA
Staff is appropriately trained in defined technical and non-technical skills, including pain management	88.64%	7.57	±1.21	46.51%	6.21	±1.99				50.00%	6.24	±1.97	NA
The number of existing theatres (excluding radiology suites, dedicated obstetric, minor operations but including day theatre) is considered adequate according to national recommendations	68.18%	6.07	±1.79	67.44%	6.65	±1.74	82.98%	6.60	±1.42	80.43%	6.98	±1.53	NA
There is an internal policy for receiving feedback from patients and caregivers, including complaints, in place.	79.55%	7.43	±1.25	72.09%	6.88	±1.68				82.61%	6.93	±1.45	NA
High-risk patients are discussed in regular speciality multidisciplinary team meetings with anaesthetic representation	85.11%	7.57	±1.50	54.35%	6.13	±1.85				61.70%	6.32	±1.75	NA

National policy for patient identification is followed, including labelling patients, re lacing labels, and using the patient's two identifiers at every stage of theprocess.	93.62%	7.74	±1.09	56.52%	6.37	±1.82				70.21%	6.64	±1.58	NA
Minimum monitoring is attached to patients before induction of anaesthesia, and their use continues until the patient has recovered from the effects of anaesthesia	95.56%	7.89	±0.96	63.04%	6.70	±1.67				74.47%	6.83	±1.60	NA
Patients are cared for on a one-to-one basis by competent and appropriately trained recovery staff until they can maintain their airway, breathing and circulation	91.30%	7.61	±0.96	54.35%	6.39	±1.70				57.45%	6.36	±1.81	NA
Patient and caregivers' education regarding their essential roles in achieving comfort, reporting pain, and adequately using the recommended analgesic methods is conducted by anaesthetists offering perioperative analgesia in collaboration with others as appropriate	89.13%	7.46	±0.96	30.43%	5.78	±1.48				27.66%	5.89	±1.34	NA
Enhanced recovery is recorded if applicable	80.43%	6.67	±1.30	47.83%	5.89	±2.11	91.49%	6.96	±1.01	59.57%	6.30	±1.82	Not scored
Whether pain is measured within the postoperative period, including predischarge and after discharge	88.89%	7.40	±1.01	58.70%	6.37	±1.73				76.60%	6.85	±1.52	NA
The patient is transferred according to recommendations, and this is done by suitably trained staff	93.48%	7.52	±0.96	46.67%	6.16	±1.47				56.52%	6.24	±1.42	NA
Patients on the enhanced recovery pathway for colonic	73.33%	6.58	±1.47	46.67%	6.18	±1.90	82.98%	6.77	±1.22	63.04%	6.65	±1.69	7

surgery are followed up for a minimum of 30 days													
A follow-up phone call within seven days of discharge is recorded	65.12%	6.14	±1.83	68.18%	6.75	±2.11	72.34%	6.23	±1.52	82.22%	7.16	±1.76	NA
The length of stay is measured	78.57%	7.10	±1.74	86.05%	7.58	±1.50							NA
Reports on the quality and costs of healthcare services are disseminated by the hospital to its community				51.22%	6.12	±2.38	60.47%	6.40	±1.68	72.73%	6.64	±2.13	NA
Patient satisfaction	80.85%	7.45	±1.33	45.65%	6.46	±1.36				42.55%	6.46	±1.36	NA
Patient well-being	80.85%	7.43	±1.04	41.30%	5.98	±1.48				36.17%	5.94	±1.21	NA
Patient comfort	80.85%	7.19	±1.12	43.48%	6.02	±1.55				40.43%	6.02	±1.23	NA
Perioperative thermal comfort	72.34%	7.13	±1.39	50.00%	6.15	±1.63	78.72%	7.19	±1.32	63.83%	6.38	±1.33	7
Health-related quality of life	87.23%	7.64	±1.12	28.26%	5.65	±1.75				25.53%	5.77	±1.57	NA
Caregiver satisfaction	72.34%	7.17	±1.26	39.13%	6.11	±1.58	74.47%	7.11	±1.20	42.55%	6.28	±1.33	4
Pain¶	87.23%	7.85	±1.18	76.09%	7.00	±1.27	83.33%	7.12	±1.07	61.54%	6.49	±1.19	9
Pain at rest¶	80.00%	7.44	±1.26	75.56%	6.98	±1.35							NA
Pain on movement¶	77.78%	7.33	±1.30	70.45%	6.86	±1.44							NA
Impact of pain on activity	80.00%	7.38	±1.20	51.11%	6.38	±1.72				53.19%	6.34	±1.55	NA
Pain-associated fear	68.18%	6.95	±1.37	42.22%	6.11	±1.66	72.34%	6.94	±1.25	42.55%	6.09	±1.46	NA
Perioperative anxiety	77.27%	7.23	±1.32	43.18%	5.91	±1.63				55.56%	6.07	±1.54	NA
Absence of falls following surgery	83.33%	7.50	±1.41	71.43%	7.00	±1.57				82.61%	7.07	±1.33	NA
Perioperative hypothermia	83.33%	7.52	±1.35	66.67%	6.95	±1.52	93.62%	7.77	±1.16	84.78%	7.15	±1.29	NA
Perioperative hypoglycaemic events	88.10%	7.69	±1.30	70.73%	7.02	±1.47				86.67%	7.22	±1.28	NA
Transfused patients	82.93%	7.68	±1.18	85.00%	7.48	±1.50							NA
Venous thromboembolism prophylaxis	92.86%	7.74	±0.95	75.00%	7.15	±1.32							NA
Anticoagulation therapy	90.48%	7.43	±1.08	77.50%	7.08	±1.47							NA
Troponin assessment proportion	65.85%	6.54	±1.65	60.00%	6.13	±1.78	74.47%	6.49	±1.58	64.44%	6.09	±1.69	NA
CHA2DS2-VASc score	60.53%	5.87	±2.06	56.41%	5.82	±2.01	67.39%	5.87	±1.99	62.22%	5.69	±2.02	NA
Financial cost of in-hospital care¤	55.00%	6.28	±1.96	33.33%	5.21	±2.29							NA

* Measure added after round 1 of the eDelphi Technique based on the experts' suggestions.

¤ Measure removed after round 1 of the eDelphi Technique based on the experts' suggestions.

¶ Measure merged after round 1 of the eDelphi Technique based on the experts' suggestions.
^ Despite a subgroup score of 4, experts agreed in plenary that the measure was important to include in the preliminary final list.
