# Supplementary material for: Core Measure Set for Patient Safety in Perioperative Care: A Clinical Practice-Oriented Consensus Study
Source: Int J Public Health. 2026 Mar 2;71:1609159. doi: 10.3389/ijph.2026.1609159 (PMC12989447; doi:10.3389/ijph.2026.1609159)
Supplement: Supplementary file 4 [file DataSheet1.pdf]

International Journal of Public Health

Title: **Core Measure Set for Patient Safety in Perioperative Care: A Clinical Practice-Oriented Consensus Study**

Supplementary file S1 - Search strategies used in of the umbrella review "Umbrella review of measures for patient safety in perioperative care" (European Union, 2022)

Search terms blocks:

1. Core Outcome and Measures Set
2. Perioperative care
3. Patient Safety
4. Systematic Review

PubMed (06/10/2022, n= 1234)

(("core outcome set*"[All Fields] OR ("Endpoint Determination"[MeSH Terms] OR "Patient Reported Outcome Measures"[MeSH Terms] OR "patient reported experience measures"[Title/Abstract] OR "quality indicators, health care"[MeSH Terms] OR "Health Status Indicators"[MeSH Terms] OR "Treatment Outcome"[MeSH Terms] OR ((("outcome and process assessment, health care"[MeSH Terms] OR "outcome assessment, health care"[MeSH Terms] OR "Quality of Life"[MeSH Terms] OR "Patient Satisfaction"[MeSH Terms] OR "Patient Comfort"[MeSH Terms] OR "Patient Outcome Assessment"[MeSH Terms] OR "process assessment, health care"[MeSH Terms]) AND "outcome*"[Title/Abstract]) OR "endpoint*"[Title/Abstract] OR "Assessment"[Title/Abstract] OR "measure*"[Title/Abstract] OR "indicator*"[Title/Abstract]))) AND ("Consensus"[MeSH Terms] OR "Delphi Technique"[MeSH Terms] OR "consens*"[Title/Abstract] OR "delphi*"[Title/Abstract] OR "standardiz*"[Title/Abstract] OR "recommend*"[Title/Abstract]) AND ("Perioperative Care"[MeSH Terms] OR "Preoperative Care"[MeSH Terms] OR "Postoperative Care"[MeSH Terms] OR "Intraoperative Care"[MeSH Terms] OR (("perioperative*"[Title/Abstract] OR "preoperative*"[Title/Abstract] OR "intraoperative*"[Title/Abstract] OR "postoperative*"[Title/Abstract]) AND ("Period"[Title/Abstract] OR "Medicine"[Title/Abstract] OR "complication*"[Title/Abstract] OR "event*"[Title/Abstract] OR "management*"[Title/Abstract] OR "time"[Title/Abstract] OR "phase*"[Title/Abstract]) AND "surgical procedures, operative"[MeSH Terms])) AND ("Patient Safety"[MeSH Terms] OR ("patient s"[All Fields] OR "patients"[MeSH Terms]

OR "patients"[All Fields] OR "patient"[All Fields] OR "patient"[Title/Abstract]) OR "Safety Management"[MeSH Terms] OR "Medical Audit"[MeSH Terms] OR "near miss, healthcare"[MeSH Terms] OR "Patient Harm"[MeSH Terms] OR "Medical Errors"[MeSH Terms] OR (("safe*"[Title/Abstract] OR "error*"[Title/Abstract] OR "Adverse"[Title/Abstract]) AND ("quality assurance, health care"[MeSH Terms] OR "Quality Improvement"[MeSH Terms] OR "Quality of Health Care"[MeSH Terms] OR "Risk Management"[MeSH Terms])))) AND "systematic review"[Filter]

Scopus (09/10/2022, n= 120)
("core outcome* set*" OR (("patient reported outcome measures" OR "patient reported experience measures" OR "health care quality indicators" OR "health status indicators" OR "treatment outcome") OR (((health care outcome and process assessment) OR (health care outcome assessment) OR "Quality of Life" OR "Patient Satisfaction" OR "Patient Comfort" OR "Patient Outcome Assessment" OR "health care process assessment") AND (TITLE-ABS(outcome*) OR TITLE-ABS(endpoint*) OR TITLE-ABS(Assessment) OR TITLE-ABS(measure*) OR TITLE-ABS(indicator*))) AND ("Delphi Technique" OR TITLE-ABS(consens*) OR TITLE-ABS(delphi*) OR TITLE-ABS(standardiz*) OR TITLE-ABS(recommend*)))) AND ((TITLE-ABS(perioperative*) OR TITLE-ABS(preoperative*) OR TITLE-ABS(intraoperative*) OR TITLE-ABS(postoperative*)) AND ((TITLE-ABS(Period) OR TITLE-ABS(Medicine) OR TITLE-ABS(complication*) OR TITLE-ABS(event*) OR TITLE-ABS(management*) OR TITLE-ABS(time) OR TITLE-ABS(phase*)) AND "surgical procedur*")) AND ("patient safety" OR TITLE-ABS("safety management") OR TITLE-ABS("medical audit") OR TITLE-ABS("health care near miss") OR TITLE-ABS("patient harm") OR TITLE-ABS("medical error*") OR ((TITLE-ABS(safe*) OR TITLE-ABS(error*) OR TITLE-ABS(adverse)) AND (TITLE-ABS("health care quality assurance") OR TITLE-ABS("quality improvement") OR TITLE-ABS("quality of health care") OR TITLE-ABS("risk management")))) AND (LIMIT-TO (DOCTYPE,"re"))

Web of Science (Core Collection) (29/12/2022, n= 206)
((ALL="core outcome set*" OR (ALL="Endpoint Determination" OR ALL="Patient Reported Outcome Measures" OR (TI="patient reported experience measures" OR AB="patient reported experience measures") OR ALL="quality indicators, health care" OR ALL="Health Status Indicators" OR ALL="Treatment Outcome" OR (((ALL="outcome and process assessment, health care" OR ALL="outcome assessment, health care" OR ALL="Quality of Life" OR ALL="Patient Satisfaction" OR ALL="Patient Comfort" OR ALL="Patient Outcome Assessment" OR ALL="process assessment,

health care") AND (TI=outcome* OR AB=outcome*)) OR (TI=endpoint* OR AB=endpoint*) OR (TI=Assessment OR AB=Assessment) OR (TI=measure* OR AB=measure*) OR (TI=indicator* OR AB=indicator*)))) AND (ALL=Consensus OR ALL="Delphi Technique" OR (TI=consens* OR AB=consens*) OR (TI=delphi* OR AB=delphi*) OR (TI=standardiz* OR AB=standardiz*) OR (TI=recommend* OR AB=recommend*)) AND (ALL="Perioperative Care" OR ALL="Preoperative Care" OR ALL="Postoperative Care" OR ALL="Intraoperative Care" OR (((TI=perioperative* OR AB=perioperative*) OR (TI=preoperative* OR AB=preoperative*) OR (TI=intraoperative* OR AB=intraoperative*) OR (TI=postoperative* OR AB=postoperative*)) AND ((TI=Period OR AB=Period) OR (TI=Medicine OR AB=Medicine) OR (TI=complication* OR AB=complication*) OR (TI=event* OR AB=event*) OR (TI=management* OR AB=management*) OR (TI=time OR AB=time) OR (TI=phase* OR AB=phase*)) AND ALL="surgical procedures, operative")) AND (ALL="Patient Safety" OR (ALL="patient s" OR ALL=patients OR ALL=patients OR ALL=patient OR (TI=patient OR AB=patient)) OR ALL="Safety Management" OR ALL="Medical Audit" OR ALL="near miss, healthcare" OR ALL="Patient Harm" OR ALL="Medical Errors" OR (((TI=safe* OR AB=safe*) OR (TI=error* OR AB=error*) OR (TI=Adverse OR AB=Adverse)) AND (ALL="quality assurance, health care" OR ALL="Quality Improvement" OR ALL="Quality of Health Care" OR ALL="Risk Management"))))

Embase (11/11/2022, n= 48)
#65. #21 AND #28 AND #37 AND #45 AND #46 AND #60 AND #63 AND [systematic review]/lim
#64. #21 AND #28 AND #37 AND #45 AND #46 AND #60 AND #63
#63. #61 OR #62
#62. 'risk management'/exp
#61. 'total quality management'/exp
#60. #47 OR #48 OR #49 OR #50 OR #51 OR #52 OR #53 OR#54 OR #55 OR #56 OR #57 OR #58 OR #59
#59. adverse
#58. error*
#57. safe*
#56. 'medical error'/exp
#55. 'patient harm'/exp
#54. 'near miss (health care)'/exp
#53. 'clinical audit'/exp

#52. 'safety'/exp

#51. patient*

#50. 'patient'/exp

#49. patient

#48. patients

#47. 'patient safety'/exp

#46. 'surgery'/exp

#45. #38 OR #39 OR #40 OR #41 OR #42 OR #43 OR #44

#44. phase*

#43. time

#42. management*

#41. event*

#40. complication*

#39. medicine

#38. period

#37. #29 OR #30 OR #31 OR #32 OR #33 OR #34 OR #35 OR #36

#36. postoperative*

#35. intraoperative*

#34. preoperative*

#33. perioperative*

#32. 'peroperative care'/exp

#31. 'postoperative care'/exp

#30. 'preoperative care'/exp

#29. 'perioperative care'/exp

#28. #22 OR #23 OR #24 OR #25 OR #26 OR #27

#27. recommend*

#26. standardiz*

#25. delphi*

#24. consens*

#23. 'delphi study'/exp

#22. 'consensus'/exp

#21. #1 OR #2 OR #3 OR #4 OR #5 OR #6 OR #7 OR #16 OR #17 OR #18 OR #19 OR #20

#20. indicator*

#19. measurement*

#18. assessment

#17. endpoint*

#16. #14 AND #15

#15. #8 OR #9 OR #10 OR #11 OR #12 OR #13

#14. outcome*

#13. 'patient outcome assessment'

#12. 'patient comfort'/exp

#11. 'patient satisfaction'/exp

#10. 'quality of life'/exp

#9. 'outcome assessment'/exp

#8. 'process assessment, health care'

#7. 'treatment outcome'/exp

#6. 'health status indicator'/exp

#5. 'health care quality'/exp

#4. 'patient reported experience measures'

#3. 'patient-reported outcome'/exp

#2. 'bioassay'/exp

#1. 'core outcome set'

The Cochrane Library (Cochrane Database of Systematic Reviews) (09/10/2022, n= 311)

Core Outcome Set*

MeSH descriptor: [Endpoint Determination] explode all trees

MeSH descriptor: [Patient Reported Outcome Measures] explode all trees

ti,ab Patient-Reported Experience Measures

MeSH descriptor: [Quality Indicators, Health Care] explode all trees

MeSH descriptor: [Health Status Indicators] explode all trees

MeSH descriptor: [Patient Outcome Assessment] explode all trees

MeSH descriptor: [Outcome and Process Assessment, Health Care] explode all trees

MeSH descriptor: [Outcome Assessment, Health Care] explode all trees

MeSH descriptor: [Treatment Outcome] explode all trees

MeSH descriptor: [Process Assessment, Health Care] explode all trees

MeSH descriptor: [Quality of Life] explode all trees

MeSH descriptor: [Patient Comfort] explode all trees

MeSH descriptor: [Patient Satisfaction] explode all trees

ti,ab Outcome* OR ti,ab Endpoint* OR ti,ab Assessment OR ti,ab Measure* OR ti,ab indicator*

MeSH descriptor: [Consensus] explode all trees

MeSH descriptor: [Delphi Technique] explode all trees

ti,ab "consens*"OR ti,ab "delphi*" OR ti,ab "Standardiz*"OR ti,ab "Recommend*"

#1 OR #2 OR #3 OR #4 OR #5 OR #6 OR #10

#8 OR #9 OR #12 OR #14 OR #13 OR #7 OR #11

#20 AND #15

#16 OR #17 OR #18

MeSH descriptor: [Perioperative Care] explode all trees

MeSH descriptor: [Preoperative Care] explode all trees

MeSH descriptor: [Postoperative Care] explode all trees

MeSH descriptor: [Intraoperative Care] explode all trees

MeSH descriptor: [Anesthesia Recovery Period] explode all trees

MeSH descriptor: [Surgical Procedures, Operative] explode all trees

ti,ab Perioperative*

ti,ab Preoperative*

ti,ab Intraoperative*

ti,ab Postoperative*

ti,ab Period OR ti,ab Medicine OR ti,ab Complication* OR ti,ab Event* OR ti,ab management* OR ti,ab time OR ti,ab phase*

MeSH descriptor: [Patient Safety] explode all trees

MeSH descriptor: [Safety Management] explode all trees

MeSH descriptor: [Medical Audit] explode all trees

MeSH descriptor: [Near Miss, Healthcare] explode all trees

MeSH descriptor: [Patient Harm] explode all trees

MeSH descriptor: [Medical Errors] explode all trees

MeSH descriptor: [Quality Improvement] explode all trees

MeSH descriptor: [Quality Assurance, Health Care] explode all trees

MeSH descriptor: [Risk Management] explode all trees

MeSH descriptor: [Quality of Health Care] explode all trees

MeSH descriptor: [Safety] explode all trees

MeSH descriptor: [Iatrogenic Disease] explode all trees

ti,ab Safe*

ti,ab Error*

ti,ab Adverse

patients

patient

patient s

MeSH descriptor: [Patients] explode all trees

#52 OR #49 or #50 or #51

#35 OR #36 OR #37 OR #38 OR #39

#46 OR #47 OR #48

#19 OR #21

#56 AND #18

#23 OR #24 OR #25 OR #26 OR #27 OR #28

#29 OR #30 OR #31 OR #32

#59 AND #33

#58 OR #60

#34 OR #49 OR #50 OR #51

#35 OR #36 OR #37 OR #38 OR #39

#46 OR #47 OR #48

#41 OR #42 OR #43 OR #44

#64 AND #65

#62 OR #63 OR #66

#57 AND #60 AND #67 in Cochrane Reviews

Cumulative Index to Nursing and Allied Health Literature (CINAHL) (09/10/2022, n= 92)

(("core outcome set*" OR ((MH "Endpoint Determination"+) OR (MH "Patient Reported Outcome Measures"+) OR (TI "patient reported experience measures" OR AB "patient reported experience measures") OR (MH "quality indicators, health care"+) OR (MH "Health Status Indicators"+) OR (MH

"Treatment Outcome"+) OR ((((MH "outcome and process assessment, health care"+) OR (MH "outcome assessment, health care"+) OR (MH "Quality of Life"+) OR (MH "Patient Satisfaction"+) OR (MH "Patient Comfort"+) OR (MH "Patient Outcome Assessment"+) OR (MH "process assessment, health care"+)) AND (TI outcome* OR AB outcome*)) OR (TI endpoint* OR AB endpoint*) OR (TI Assessment OR AB Assessment) OR (TI measure* OR AB measure*) OR (TI indicator* OR AB indicator*)))) AND ((MH Consensus+) OR (MH "Delphi Technique"+) OR (TI consens* OR AB consens*) OR (TI delphi* OR AB delphi*) OR (TI standardiz* OR AB standardiz*) OR (TI recommend* OR AB recommend*)) AND ((MH "Perioperative Care"+) OR (MH "Preoperative Care"+) OR (MH "Postoperative Care"+) OR (MH "Intraoperative Care"+) OR (((TI perioperative* OR AB perioperative*) OR (TI preoperative* OR AB preoperative*) OR (TI intraoperative* OR AB intraoperative*) OR (TI postoperative* OR AB postoperative*)) AND ((TI Period OR AB Period) OR (TI Medicine OR AB Medicine) OR (TI complication* OR AB complication*) OR (TI event* OR AB event*) OR (TI management* OR AB management*) OR (TI time OR AB time) OR (TI phase* OR AB phase*)) AND (MH "Surgery, Operative"))) AND ((MH "Patient Safety"+) OR ("patient s" OR (MH patients+) OR patients OR patient OR (TI patient OR AB patient)) OR (MH "Safety Management"+) OR (MH "Medical Audit"+) OR (MH "near miss, healthcare"+) OR (MH "Patient Harm"+) OR (MH "Medical Errors"+) OR (((TI safe* OR AB safe*) OR (TI error* OR AB error*) OR (TI Adverse OR AB Adverse)) AND ((MH "quality assurance, health care"+) OR (MH "Quality Improvement"+) OR (MH "Quality of Health Care"+) OR (MH "Risk Management"+))))) AND "systematic review[Filter]"

COMET Initiative Database (09/10/2022, n= 50)

Perioperative care

Preoperative care

Postoperative care

Intraoperative care

Perioperative medicine

Perioperative management

Perioperative time

Perioperative phase

Perioperative period

Perioperative event

Perioperative complication

Patient safety

Safety management

Medical audit

Healthcare near miss

Patient harm

Medical errors

Quality improvement

Quality assurance

Risk management
